# Aqueous alkaline phosphate facilitates the non-exchangeable deuteration of peptides and proteins†

**DOI:** 10.1039/d3ra08636d

**Published:** 2024-03-08

**Authors:** Tingting Zhang, Zhixiong Jin, Heng Zhao, Can Lai, Zheyi Liu, Pan Luo, Zhe Dong, Fangjun Wang

**Affiliations:** a CAS Key Laboratory of Separation Sciences for Analytical Chemistry, State Key Laboratory of Molecular Reaction Dynamics, Dalian Institute of Chemical Physics, Chinese Academy of Sciences Dalian 116023 China wangfj@dicp.ac.cn; b University of Chinese Academy of Sciences Beijing 100049 China; c Department of Chemistry, Zhejiang University Hangzhou 310027 China; d Institute of Advanced Science Facilities Shenzhen 518000 China; e Shenzhen Grubbs Institute and Department of Chemistry, Guangdong Provincial Key Laboratory of Catalysis, Southern University of Science and Technology Shenzhen 518000 China

## Abstract

The incorporation of deuterium into peptides and proteins holds broad applications across various fields, such as drug development and structural characterization. Nevertheless, current methods for peptide/protein deuteration often target exchangeable labile sites or require harsh conditions for stable modification. In this study, we present a late-stage approach utilizing an alkaline phosphate solution to achieve deuteration of non-exchangeable backbone sites of peptides and proteins. The specific deuteration regions are identified through ultraviolet photodissociation (UVPD) and mass spectrometry analysis. This deuteration strategy demonstrates site and structure selectivity, with a notable affinity for labeling the α-helix regions of myoglobin. The deuterium method is particularly suitable for peptides and proteins that remain stable under high pH conditions.

## Abstract 2

The incorporation of deuterium into peptides and proteins holds broad applications across various fields, such as drug development and structural characterization.

## Graphical abstract

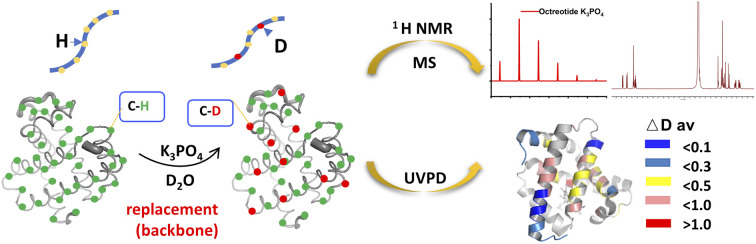

## 1 Introduction

Deuterium, a stable hydrogen isotope with one proton and one neutron in its nucleus,^1^ plays a pivotal role in pharmaceutical development^2–4^ and serves as a valuable tracer in life science research.^5,6^ In recent years, there has been rapid progress in the methods for preparing deuterium-labeled amino acids,^7^ peptides, and proteins.^5,6,8^ The incorporation of deuterium into peptides/proteins can be achieved either through non-stable hydrogen-deuterium exchange (HDX) or *via* multistep peptide synthesis to introduce stable deuterium. However, deuterated peptide/protein synthesis through chemical or biosynthetic approaches using commercially available monomers is time- and resource-consuming.^9–13^ Therefore, the late-stage introduction of stable deuterium into peptides/proteins has been extensively investigated.^14^

**Fig. 1:**
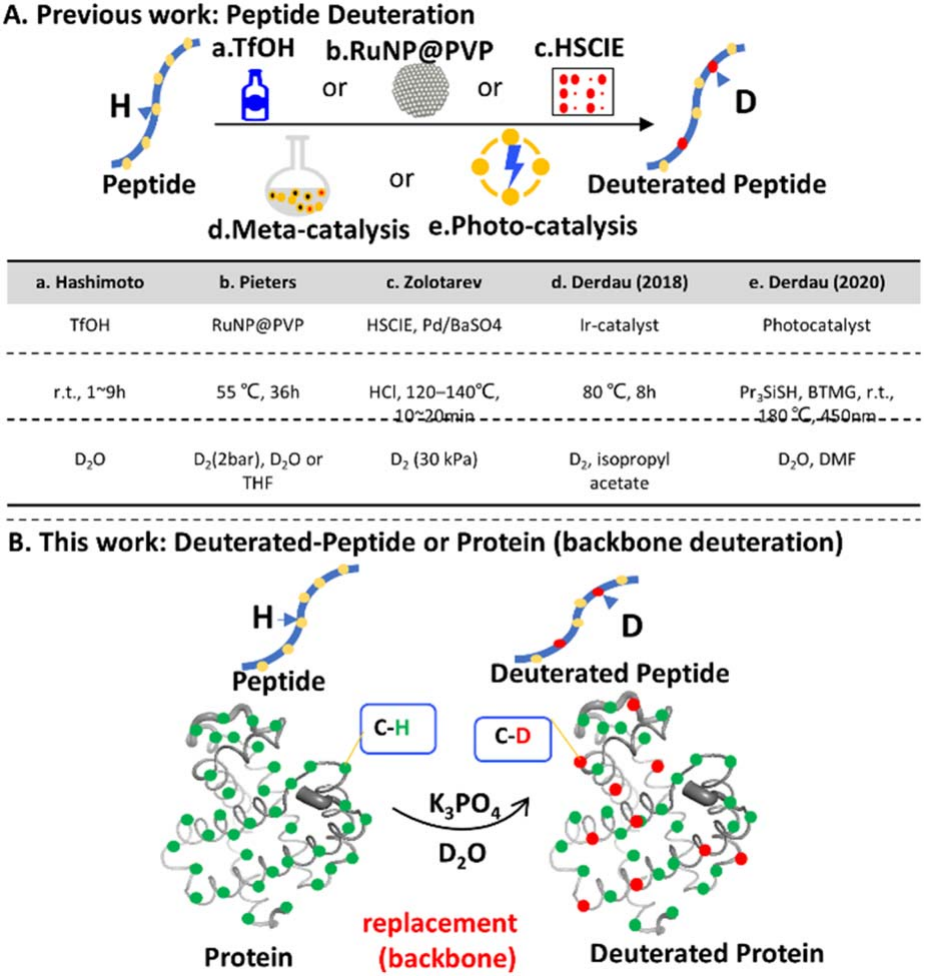
Schematic diagram of strategies for late-stage peptide/protein deuteration. (A) Peptide deuteration methods including (a) TfOH (aromatic residue of peptides), (b) a poly-vinyl-pyrrolidone matrix (RuNP@PVP) catalysed C–H activation and deuteration, (c) high-temperature solid phase catalytic isotope exchange (HSCIE), (d) metal Ir catalysis (di- and tripeptides), and (e) photo redox-initiated hydrogen atom transfer (HAT). (B) This work utilizes alkaline phosphate solution to achieve stable deuteration of peptide/protein directly (optimal deuteration conditions identified as 100 mM K_3_PO_4_ with pH 12.7 at 25 °C).

Until now, late-stage methods for the preparation of deuterated peptides primarily include acid (TfOH)-mediated peptide deuteration,^15^ catalytic hydrogen isotope exchange (HIE) reactions facilitated by metal ruthenium nanoparticles,^16^ transition-metal-catalysed HIE reactions involving high-temperature solid phase catalytic isotope exchange (HSCIE),^17^ and the utilization of metal iridium catalysts.^18^ Furthermore, an additional technique involves exploiting the lipid solubility of photo-catalysts to induce photo-redox-initiated hydrogen atom transfer (HAT), as illustrated in Fig. 1A.^19^ These methods realize peptide deuteration under one or several conditions, including: (1) D_2_ gas as a deuterium source; (2) temperature above 50 °C and D_2_ gas pressure >1 bar; (3) additional additives or acids (TfOH); (4) organic solvents; and (5) catalytic systems such as solid phase catalytic isotope exchange (HSCIE) and catalysis involving iridium (Ir) metal (Fig. 1A). However, these conditions are largely bio-incompatible and cannot be used in biosystems. There are few deuteration approaches that are compatible with biomolecules,^6^ except non-stable HDX for labile hydrogen.^20^ Therefore, an efficient and convenient method for peptide/protein deuteration using a non-toxic and aqueous solution is urgently needed.

Water-soluble phosphate salts are widely utilized in biocompatible analysis of protein samples. In this study, we discovered peptide backbone deuteration could be realized directly in an alkaline aqueous phosphate solution with optimal pH range 12–13 (Fig. 1B). The main deuteration sites was located at the backbone of peptides. Subsequently, we employed this method for protein deuteration and the specific deuteration regions were determined by ultraviolet photodissociation (UVPD) and mass spectrometry (MS) analysis. We observed the deuterium was predominantly incorporated in the α-helix regions of myoglobin (Mb). This deuterium method is applicable to peptides and proteins that remain stable at high pH values.

## 2 Materials and methods

### 2.1 Chemicals and reagents

Reagents were purchased from commercial suppliers and used without further purification unless otherwise noted. D_2_O was purchased from Sigma-Aldrich. C_18_ AQ beads (5 μm, 120 Å) were purchased from Sunchrom (Friedrichsdorf, Germany). The deionized water used in the following experiments was purified by a Mill-Q pure water system (Millipore Inc., Milford, MA). High-performance liquid chromatography (HPLC) grade methanol and acetonitrile were purchased from Merck (Darmstadt, Germany). Myoglobin, (d,l)-tryptophan (Trp, W), (l)-phenylalanine (Phe, F), (l)-leucine(Leu, L), (l)-isoleucine(Ile, I), (l)-cysteine (Cys, C), methionine (Met, M), (l)-proline (Pro, P), (l)-glutamine (Gln, Q), formic acid (FA), trifluoroacetic acid (TFA), were purchased from Sigma Aldrich (St. Louis, MO). Standard peptides (GGGGFG, GGGGYG, GGGGWG, GGGGHG, GGGGLG, GGGGIG, GGGGMG, GGGGMG, GGGGPG, GGGGCG, GGGGQG, GGGGKG, GGGGRG) were synthesized by China Peptides Co., Ltd (Shanghai, China).

### 2.2 Sample preparation

The peptide sample (100 μM) was dissolved in deuterium oxide (D_2_O, 100 μL) with K_3_PO_4_ (100 mM, pH 12.7) at room temperature (approximately 25 °C) for 2 h (or 8 h). Then, the reaction was terminated by adjusting the pH to 7.0 with phosphoric acid. The mixture was lyophilized in a 600 μL centrifuge tube, re-dissolved into water, and desalted by using C_18_ tip column (5 μm, 120 Å). The resulting sample solution was further diluted and subjected to centrifugation (160 000*g*) for 5 min. A small aliquot of the supernatant was transferred to the sample vial for subsequent HPLC-MS analysis.

For ^1^H NMR characterization, 1 mg standard peptide was dissolved into 1 mL D_2_O with 2 M K_3_PO_4_ at room temperature (approximately 25 °C) for 8 h. Then, the reaction was terminated by adjusting the pH to 7.0 with phosphoric acid and desalted using C_18_ Extraction Cartridges (Oasis HLB 1cc, 10 mg). The mixture was then lyophilized and re-dissolved into D_2_O.

Myoglobin was dissolved in 1 mM ammonium acetate D_2_O with a concentration of 100 μM as a stock solution. An aliquot of 10 μL was mixed with 100 μL deuterium oxide (D_2_O) with K_3_PO_4_ (100 mM, pH 12.7), deoxygenated under argon protection, and kept under 37 °C water bath for 2 h. Then, the solution was exchanged to 100 mM ammonium acetate aqueous solution and replicated 6 times to remove K_3_PO_4_ and D_2_O completely by ultrafiltration (3 kD Amicon Ultra-4 Centrifugal Filter, Merck Millipore). The procedures of control protein sample preparation were identical to the deuterated protein except that K_3_PO_4_ (100 mM) was not added.

### 2.3 Analytical method

#### 2.3.1 Characterization of peptide sample through HPLC-MS and ^1^H NMR

##### 2.3.1.1 ^1^H NMR

^1^H NMR spectra were acquired using a JNM-ECZL400S-400 MHz spectrometer and an AVANCE III HD 700 MHz spectrometer. Proton chemical shifts are reported in ppm (*δ*) relative to the solvent resonance (D_2_O, *δ* 4.79 ppm). Data are reported in the following format: chemical shift (*δ* ppm), multiplicity (s = singlet, d = doublet, t = triplet, q = quartet, m = multiplet, dd = doublet of doublets, dt = doublet of triplets, br = broad), coupling constant (Hz), and integration. All NMR spectra were recorded at room temperature (approximately 25 °C).

##### 2.3.1.2 HPLC-MS

The HPLC-MS or direct MS analyses of peptide samples were conducted using the Vanquish Flex HPLC system coupled with Exactive Plus EMR MS (Thermo). The LC separation employed a mobile phase A of 0.1% formic acid (FA) aqueous solution and a mobile phase B of 0.1% FA–acetonitrile solution. Peptide samples re-dissolved into 0.1% FA (v/v) aqueous solution at a concentration of 0.1 μg μL^−1^ were processed on a capillary HPLC column system, comprising a trap column (30 mm length × 150 μm i.d., C_18_, 5 μm particle size, 120 Å pore diameter) and an analysis column (150 mm length × 75 μm i.d., C_18_, 5 μm particle size, 120 Å pore diameter) using a constant flow rate of 0.1 μL min^−1^. Samples were automatically injected (10 μL) with 5% buffer B, separation gradient increased from 5 to 35% buffer B in 10 min, flushed with 80% buffer B for 5 min, and then changed to 5% buffer B with 2 min.

A Full MS scan was conducted within the *m*/*z* range of 400–1000, employing a mass resolution of 70 000 (Exactive Plus EMR). The parameters for Exactive Plus EMR were configured as follows: ion transfer capillary at 275 °C, electrospray voltage set to 1.8 kV, and a full MS scan covering 400 to 1000 *m*/*z* with a resolution of 70 000. All MS data were acquired using the Exactive Plus EMR instrument, and subsequent data processing was carried out with an Xcalibur 2.2 (Thermo Scientific).

##### 2.3.1.3 Ultraviolet photodissociation (UVPD) and MS analysis

The deuterated Mb solution was directly infused into mass spectrometer using Static Source Glass (Static Source). Samples were loaded into an in-house pulled glass capillary (ITEM, BF 100-58-10), a 0.368 mm diameter platinum wire was inserted into the back of the capillary to provide the spray voltage. Nano-electrospray ionization (nESI) was employed, and samples were sprayed at concentration ranging from 5 to 20 μM. The spray voltage was set to 0.9 kV. All full MS and MS/MS datasets were acquired on the Orbitrap Fusion Lumos Tribrid mass spectrometer (Thermo Fisher, San Jose, CA, USA) equipped with a 193 nm ArF excimer laser as described in our previous works.^21,22^ The full mass spectrum of Mb sample was obtained with a mass resolution of 120 000. Deuterated Mb ions with a charge state of +8 were isolated with an isolation width of 3 *m*/*z* (±1.5 *m*/*z*) and subjected to 5 ns single pulse (1.0 mJ) of 193 nm laser irradiation (1.0 mJ) for UVPD analysis. The fragment mass spectra were collected by averaging 200 transients.

### 2.4 Data analysis

The raw data files were converted to mzML format using MSConvert.^23^ The masses of UVPD fragments were compared to a custom database containing the monoisotopic mass of a, *a* + 1, *b*, *c*, *x*, *x* + 1, *y*, *y* − 1, *z*, and *z* + 1 ions with a mass tolerance of ±5 ppm. This comparison was executed through custom R scripts and subsequently analysed in conjunction with the ProSightPC^24^ software package. Fragmentation tolerance was subsequently adjusted to match the total deuterium count in Mb. Finally, manual verification and comparison of mass differences were conducted between the deuterated Mb and control Mb MS/MS spectra for both deuterated and un-deuterated *m*/*z* centroid peaks. All the data processing procedures were performed using the R platform (version 4.0.4) using the R Studio graphical interface (ESI Fig. S1†).

## 3 Results and discussion

### 3.1 K_3_PO_4_ facilitates nonexchangeable CH → CD of hexapeptides and octreotide

**Fig. 2:**
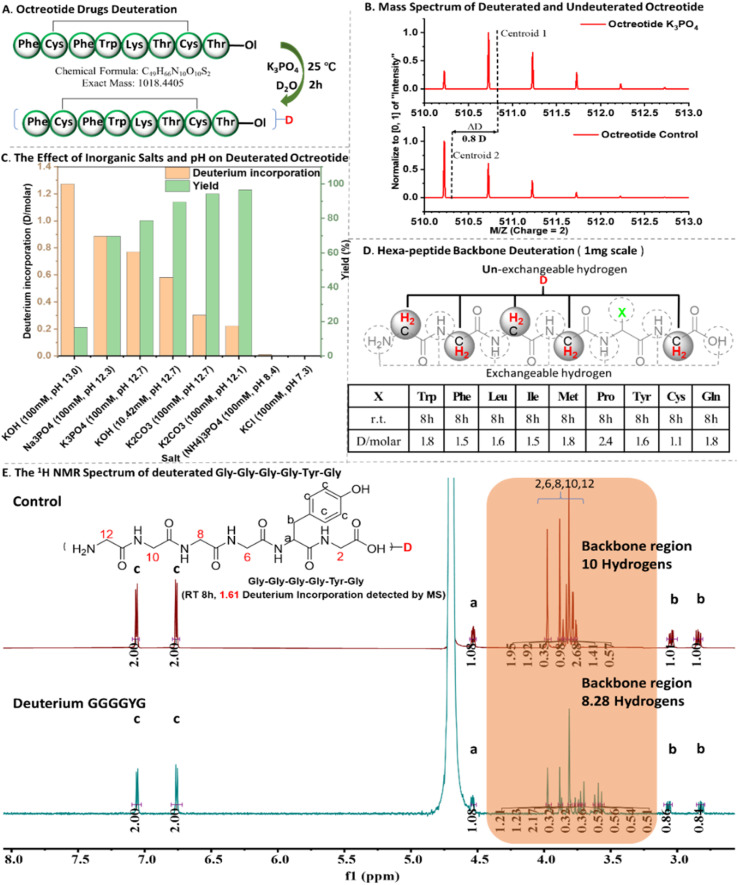
The illustration of peptide deuteration. (A) Octreotide deuteration; (B) mass spectrum of deuterated and un-deuterated octreotide; (C) the effect of inorganic salts and pH value on octreotide deuteration; (D) hexa-peptide backbone deuteration (1 mg scale); (E) the comparison of ^1^H NMR spectrum of deuterated Gly–Gly–Gly–Gly–Tyr–Gly (GGGGYG) and un-deuterated GGGGYG (other hexa-peptides ^1^H NMR spectrums are provided in ESI.†).

At first, we conducted deuteration experiments on octreotide using different kinds of aqueous salts and pH values (Fig. 2A–C). Our investigation aimed to determine the optimal conditions for octreotide deuteration, considering factors such as salt concentration, pH, peptide concentration, reaction time, and temperature (ESI Fig. S3†). The outcomes revealed a high deuterium incorporation rate (0.8–0.9 D per molecule) in alkaline phosphate solution with pH 12–13 and phosphate concentration 50–100 mM (Fig. 2C). As shown in Fig. 2C, K_3_PO_4_ (pH = 12.7) and Na_3_PO_4_ (pH = 12.3) groups exhibit both high levels of deuteration rates (0.8 and 0.9 D per molecule) and yields (78% and 81%). Although the KOH group (pH = 13.0) has the highest deuteration level (1.3 D per molecule), its deuteration yield is the lowest (18%). In contrast, KOH group (pH = 12.6), with a slightly lower pH, shows lower deuteration level but has relatively higher yield (89%). Additionally, the carbonate solution with pH 12.1–12.6 displayed a low deuterium incorporation rate (0.2–0.3 D per molecule). Thus, this peptide deuteration process is contingent on both pH and phosphate levels, with the optimal deuteration conditions identified as 100 mM K_3_PO_4_ with pH 12.7 at 25 °C.

**Fig. 3:**
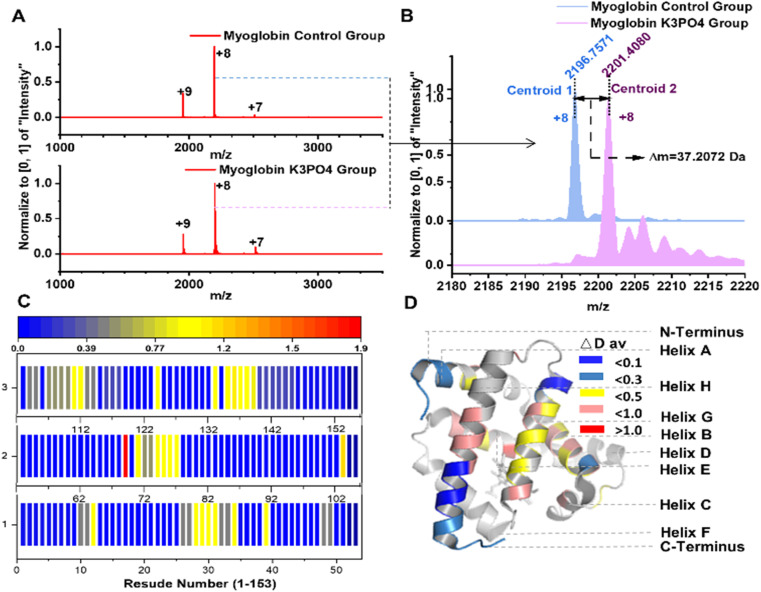
Mb deuteration with alkaline phosphate solution. (A) Native MS characterization of control and deuterated Mb samples with charge states distribution; (B) the mass shift of the Mb with or without deuteration; (C) the residue deuteration rates of Mb in UVPD analysis; (D) visualization of holo-Mb deuteration in different regions (PDB: 1WLA).

With the optimal deuteration conditions, we examined a series of standard hexapeptides GGGGXG (X, representing different amino acids except for Gly) (Fig. 2D). Liquid chromatography-mass spectrometry (LC-MS) analysis revealed the deuterium incorporation rates as follows: GGGGPG (2 h, 0.8; 8 h, 2.4 D per molecule) > GGGGWG, GGGGMG, GGGGYG, GGGGFG, GGGGQG (2 h, 0.4–0.7; 8 h, 1.6–1.8 D per molecule) > GGGGLG, GGGGIG, GGGGCG (2 h, 0.4; 8 h, 1.1–1.5 D per molecule) (Fig. 2D and ESI Fig. S3 and S4†). We hypothesize that deuterium incorporation is more likely to take place at sites where both the amino and carboxyl groups are involved in forming amide bonds and have minimal steric hindrance, facilitating nucleophilic deuterium substitution reactions (Fig. 3). To investigate the deuteration sites either at peptide backbone^16,18^ or at residue side chains,^15,17,19,25–31^^1^H NMR characterization was further performed. In the ^1^H NMR spectrum, the backbone hydrogen signals of the deuterated peptide exhibited significant distinctions compared to the control group, while the peptide side-chain signals showed minimal change (Fig. 2E and ESI Fig. S5†).^32^ Thus, the deuterated sites were stable and located at the backbone of standard hexapeptides.

### 3.2 Probing myoglobin H → D sites with aqueous alkaline phosphate

Myoglobin (Mb) is a well-studied protein with a well-definite structure.^33–35^ We further conducted deuteration of Mb with identical reaction conditions as described above. Native MS characterization indicated that 37 non-exchangeable hydrogen atoms of Mb were deuterated, and the protein charge state distribution demonstrated the relatively compact structure of Mb could be still retained after deuteration^35^ (Fig. 3A and B). Subsequently, UVPD and MS/MS analysis were applied to probe the Mb deuteration sites.^21,22^ The results revealed that most of the non-exchangeable deuterium incorporations were distributed in the core of A, B, C, G, H, and E helix regions of Mb, especially in the sequence regions Leu135-Gly150 (7.2 deuterium atoms), Gln26-Thr34 (7.0 deuterium atoms), Thr70-Leu76 (6.1 deuterium atoms), and Ile107-Lys118 (6.0 deuterium atoms) (Fig. 3C and D). These sequence regions are distributed across the α-helical structure of Mb, playing indispensable roles in both Mb stability and structural integrity.^34–36^ We speculate that the phosphate ions may diffuse into Mb and promote the partial deuteration of Mb backbone hydrogens. Therefore, aqueous alkaline phosphate facilitates the deuteration of Mb and exhibits considerable site and structure selectivity.

### 3.3 Mechanism

**Fig. 4:**
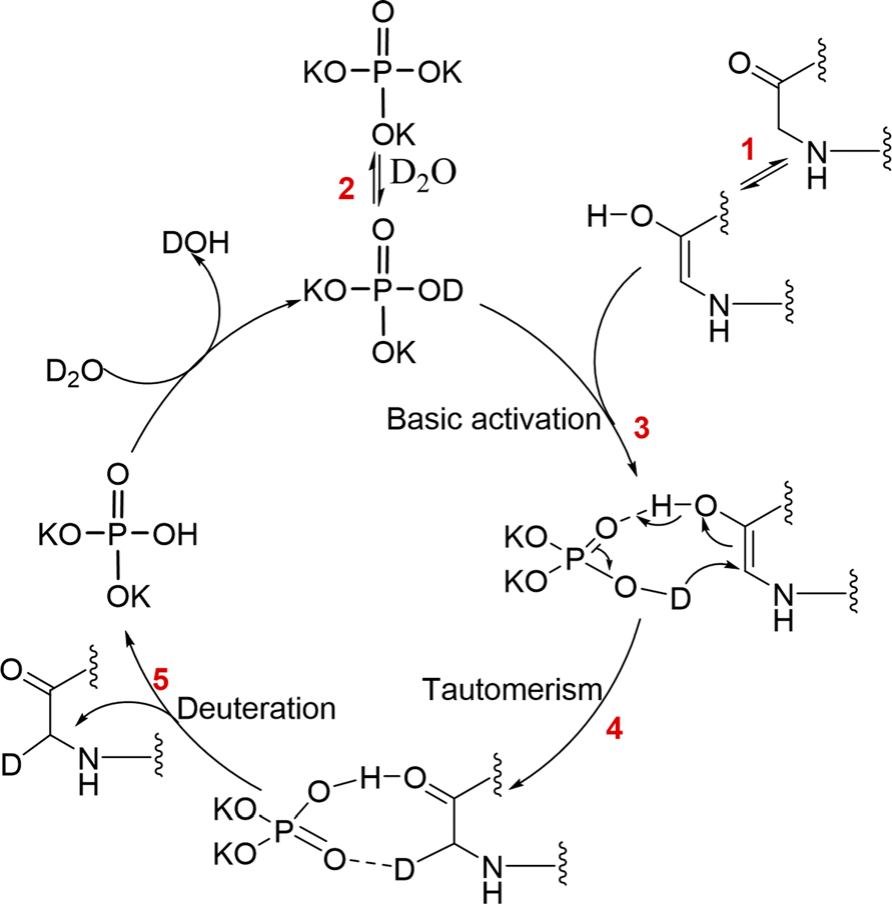
Mechanistic proposal of the interaction between potassium phosphate and peptide deuteration.

The proposed mechanism of peptide deuteration is depicted in Fig. 4. In brief, a keto–enol tautomerism equilibrium exists in the peptide at pH 12–13 (step 1).^37,38^ In the presence of K_3_PO_4_ and D_2_O, deuterated K_3_PO_4_, referred to as K_2_DPO_4_, is formed (step 2). This enables K_2_DPO_4_ to bind to octreotide,^39^ functioning as a more stable hydrogen-bond donor. It interacts with the carbon atom in the amide bond, forming a reversible chemical adduct (step 3). This adduct can undergo multiple deuteration events in the backbone when the pH value exceeds 12. Additionally, a keto–enol tautomerism equilibrium is existed in the peptide with phosphate salts (step 4), ultimately leading to the deuteration of non-exchangeable backbone sites of peptides (step 5).

## 4 Conclusion

In conclusion, we introduce a new alternative for the deuteration of peptides/proteins by using alkaline phosphate solution with pH 12–13 directly. The non-exchangeable hydrogen atoms on peptide/protein backbones can be efficiently deuterated. However, this deuterium method also has some shortcomings, including high pH values, an inability to quantify deuterium, and the potential presence of isomers. This method may affect the high-order structures and biological activity of pH sensitive proteins. Therefore, this deuteration method is more proper to proteins that can tolerate high pH environments or peptide drugs with less ordered structures. Nonetheless, this approach offers a straightforward and gentle alternative to existing techniques, paving the way for the preparation of deuterated peptides/proteins.

## Author contributions

F. W., T. Z. conceived the work and designed the experiments; T. Z. performed the deuterated sample and MS characterization experiments; T. Z., Z. L., Z. J., P. L., and C. L. performed data analysis; T. Z., H. Z. participated in protein sample preparation; Z. D. participated in NMR analysis; T. Z. and F. W. wrote the paper, T. Z., F. W., and revise the paper.

## Conflicts of interest

There are no conflicts to declare.

## Supplementary Material

RA-014-D3RA08636D-s001
