# Supplementary material for: Aqueous alkaline phosphate facilitates the non-exchangeable deuteration of peptides and proteins
Source: RSC Adv. 2024 Mar 8;14(12):8075–80. doi: 10.1039/d3ra08636d (PMC10921277; doi:10.1039/d3ra08636d)
Supplement: RA-014-D3RA08636D-s001 [file RA-014-D3RA08636D-s001.pdf]

Electronic Supplementary Material (ESI) for RSC Advances.
This journal is © The Royal Society of Chemistry 2024

Aqueous Alkaline Phosphate Facilitates the Non-exchangeable Deuteration of Peptides and Proteins

Tingting Zhang,[ab] Zhixiong Jin,[ac] Heng Zhao, [a] Can Lai,[ab] Zheyi Liu,[a] Pan Luo,[d] Zhe Dong,[e] and Fangjun Wang [*ab]

a CAS Key Laboratory of Separation Sciences for Analytical Chemistry, State Key Laboratory of Molecular Reaction Dynamics, Dalian Institute of Chemical Physics, Chinese Academy of Sciences, Dalian 116023, China.

b University of Chinese Academy of Sciences, Beijing 100049

c Department of Chemistry, Zhejiang University, Hangzhou 310027, China

d Institute of Advanced Science Facilities, Shenzhen 518000, China

e Shenzhen Grubbs Institute and Department of Chemistry, Guangdong Provincial Key Laboratory of Catalysis, Southern University of Science and Technology, Shenzhen, China.

*Dalian Institute of Chemical Physics, Chinese Academy of Sciences, Dalian 116023, China.

Email: **wangfj@dicp.ac.cn**

Table of Contents

Part I. Data Analysis. 3

Part II. Supplementary Figures 5

Part III. Supporting Information NMR Part 8

Part IIII. Supporting Information HPLC-MS/MS Part .. 16

Part I. Data Analysis.

Peptide:

The average mass of peptide is calculated by taking the average mass of all the isotopic peaks weighted by their intensities. This means that for an isotopic peak distribution containing n peaks the average mass (M) can be found using Equation 1

Equation 1:

$$\mathrm{M}_{deuterium} = \frac{\sum_{i=1}^{n} \mathrm{I_i} \cdot M_i}{\sum_{i=1}^{n} \mathrm{I_i}}$$

$$\mathrm{M}_{un-deuterium} = \frac{\sum_{i=1}^{n} \mathrm{I_i} \cdot M_i}{\sum_{i=1}^{n} \mathrm{I_i}}$$

$$\frac{\mathrm{Deuterium}}{molecular} = (\mathrm{M}_{deuterium} - \mathrm{M}_{un-deuterium}) * charge$$

Protein:

$$\mathrm{Deuterium\ mass} = [(^{m}/_{z})_{deuterium} - (^{m}/_{z})_{undeuterium}] * z$$

where $(^{m}/_{z})_{deuterium}$ is the centroid mass of the fragment ions of interest, whereas $(^{m}/_{z})_{undeuterium}$ is the corresponding reference data for completely unlabeled samples fragment ions.

All biochemical graphs were produced using OriginPro 2019b (OriginLab) or R script.

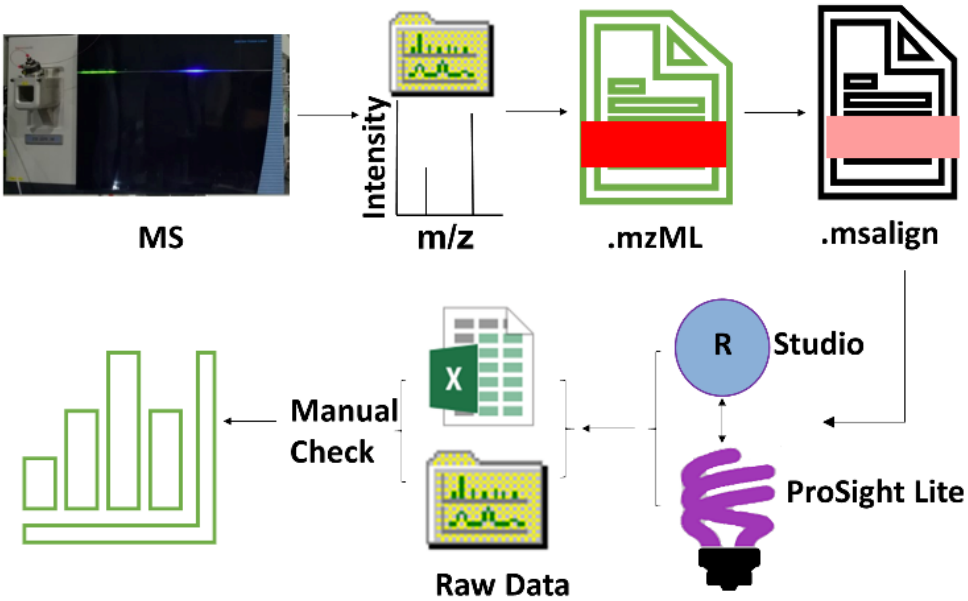

Figure S 1 Data analysis flowchart

These Mb regions include: N-terminus (Gly 1 to Ser 3), A-helix (Asp 4 to Ala 19), B-helix (Asp 20 to Gly 35), C-helix (His 36 to Lys 42), CD loop (Phe 43 to Lys 50), D-helix (Thr 51 to Ala 57), E-helix (Ser 58 to Lys 77), EF loop (Lys 78 to Glu 85), F-helix (Leu 86 to Ala 94), FG loop (Thr 95 to Ile 99), G-helix (Pro 100 to Lys 118), GH loop (His 119 to Phe 123), H-helix (Gly 124 to Gly 150), and C terminus (Phe 151 to Gly 153).

Part II. Supplementary Figures

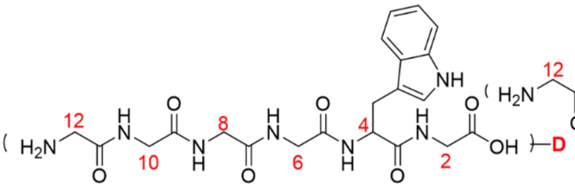

un-deuterated
Chemical Formula: $C_{21}H_{27}N_7O_7$
Exact Mass: 489.1972
Gly-Gly-Gly-Gly-Trp-Gly
(RT 8h, 1.8 Deuterium Incorporation)

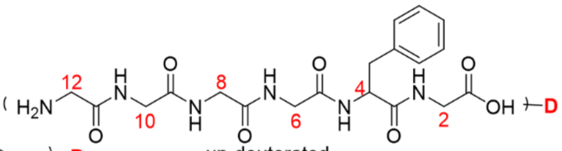

un-deuterated
Chemical Formula: $C_{19}H_{26}N_6O_7$
Exact Mass: 450.1863
Gly-Gly-Gly-Gly-Phe-Gly
(RT 8h, 1.54 Deuterium Incorporation)

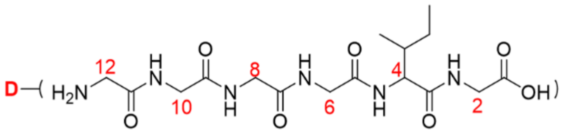

un-deuterated
Chemical Formula: $C_{16}H_{28}N_6O_7$
Exact Mass: 416.2019
Gly-Gly-Gly-Gly-Ile-Gly
(RT 8h, 1.49 Deuterium Incorporation)

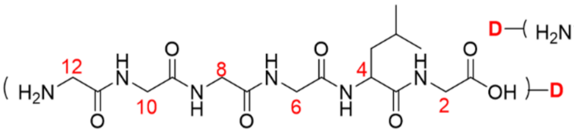

un-deuterated
Chemical Formula: $C_{16}H_{28}N_6O_7$
Exact Mass: 416.2019
Gly-Gly-Gly-Gly-Leu-Gly
(RT 8h, 1.57 Deuterium Incorporation)

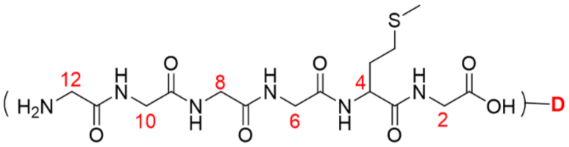

un-deuterated
Chemical Formula: $C_{15}H_{26}N_6O_7S$
Exact Mass: 434.1584
Gly-Gly-Gly-Gly-Met-Gly
(RT 8h, 1.84 Deuterium Incorporation)

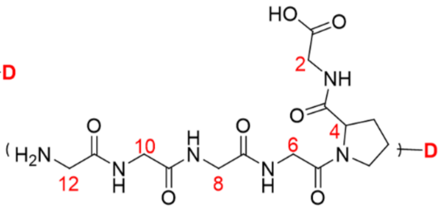

un-deuterated
Chemical Formula: $C_{15}H_{24}N_6O_7$
Exact Mass: 400.1706
Gly-Gly-Gly-Gly-Pro-Gly
(RT 8h, 2.4 Deuterium Incorporation)

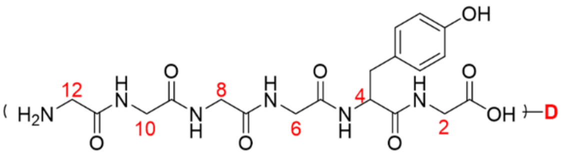

un-deuterated
Chemical Formula: $C_{19}H_{26}N_6O_8$
Exact Mass: 466.1812
Gly-Gly-Gly-Gly-Tyr-Gly
(RT 8h, 1.61 Deuterium Incorporation)

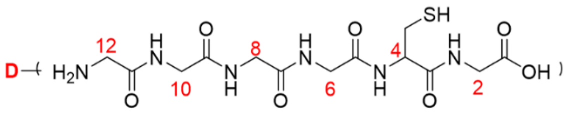

un-deuterated
Chemical Formula: $C_{13}H_{22}N_6O_7S$
Exact Mass: 406.1271
Gly-Gly-Gly-Gly-Cys-Gly
(RT 8h, 1.1 Deuterium Incorporation)

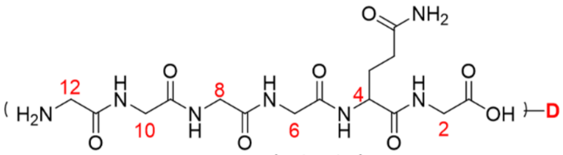

un-deuterated
Chemical Formula: $C_{15}H_{25}N_7O_8$
Exact Mass: 431.1765
Gly-Gly-Gly-Gly-Gln-Gly
(RT 8h, 1.84 Deuterium Incorporation)

Figure S2 Hexa-peptides deuteration (backbone deuteration)

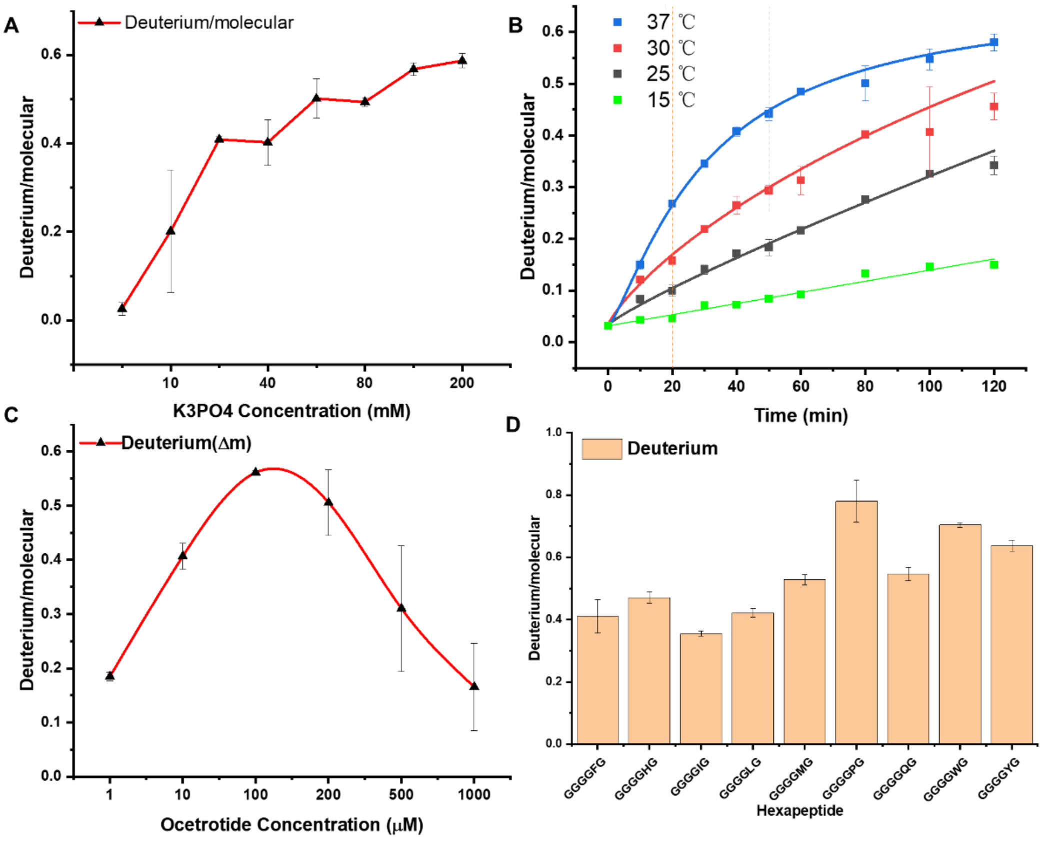

Figure S3 The investigation of optimal conditions for the deuteration of octreotide, including: A) potassium phosphate concentration, B) reaction time at 50 mM potassium phosphate condition, C) octreotide concentration (50 mM potassium phosphate), and D) the deuteration rates of standard hexapeptides at 2 hours. (Triple parallel experiments)

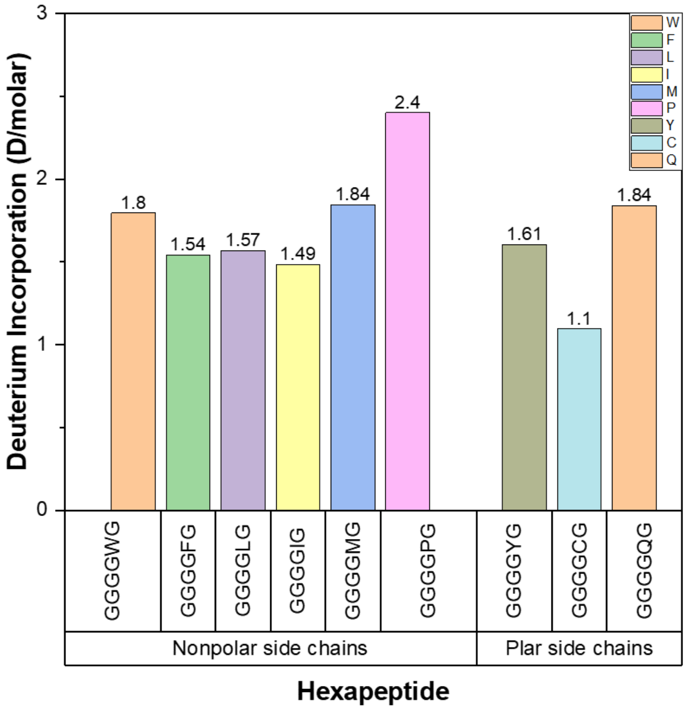

Figure S4 The deuterium incorporation of hexapeptide at 8 h (100 mM potassium phosphate).

Part III. Supporting Information NMR Part

Figure S5 ^{1}HNMR stacked spectra demonstrating the selective backbone deuteration of standard hexapeptides.

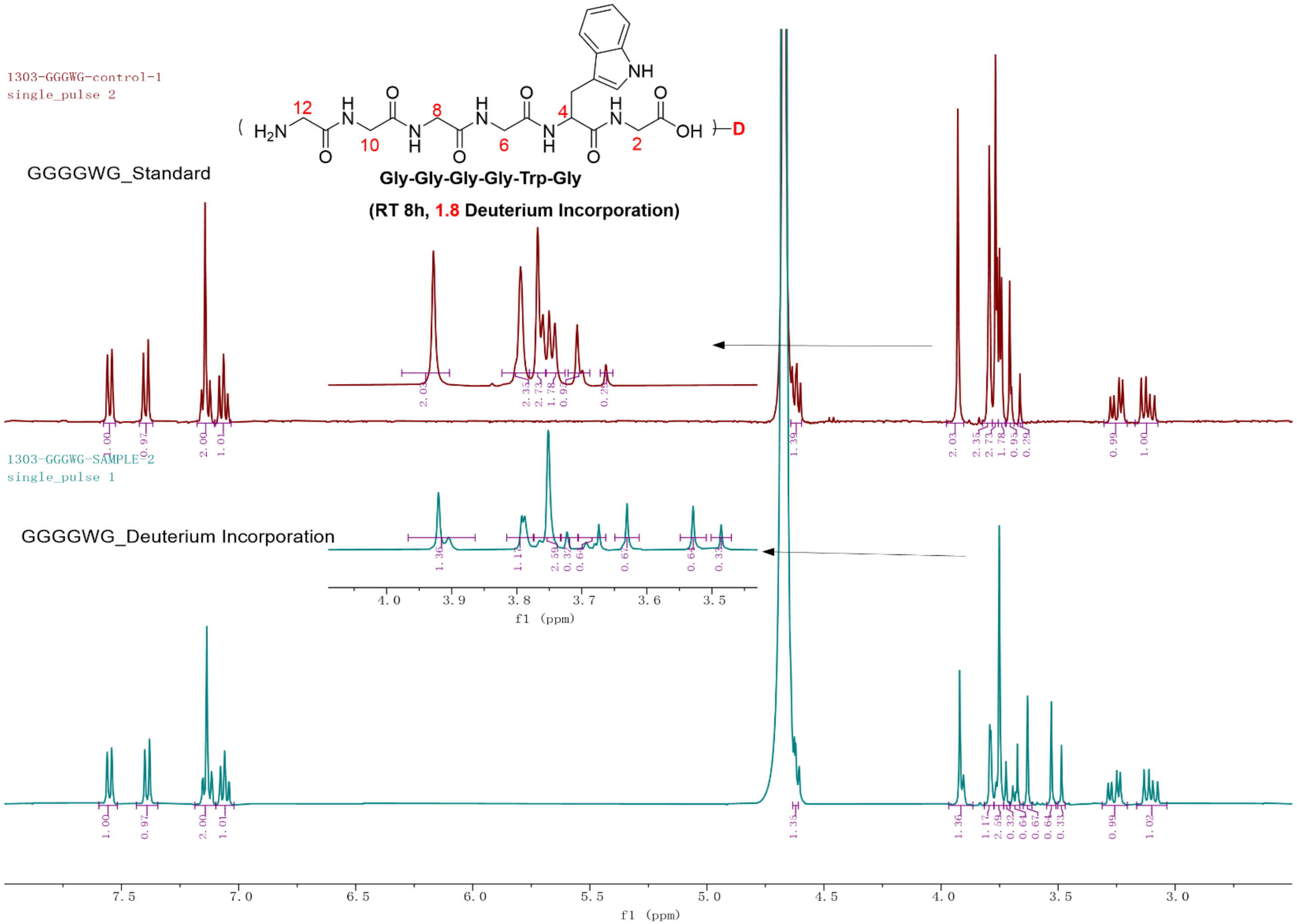
1303-GGGWG-control-1
single_pulse 2
GGGGWG_Standard
Gly-Gly-Gly-Gly-Trp-Gly
(RT 8h, 1.8 Deuterium Incorporation)
1303-GGGWG-SAMPLE-2
single_pulse 1
GGGGWG_Deuterium Incorporation
f1 (ppm)

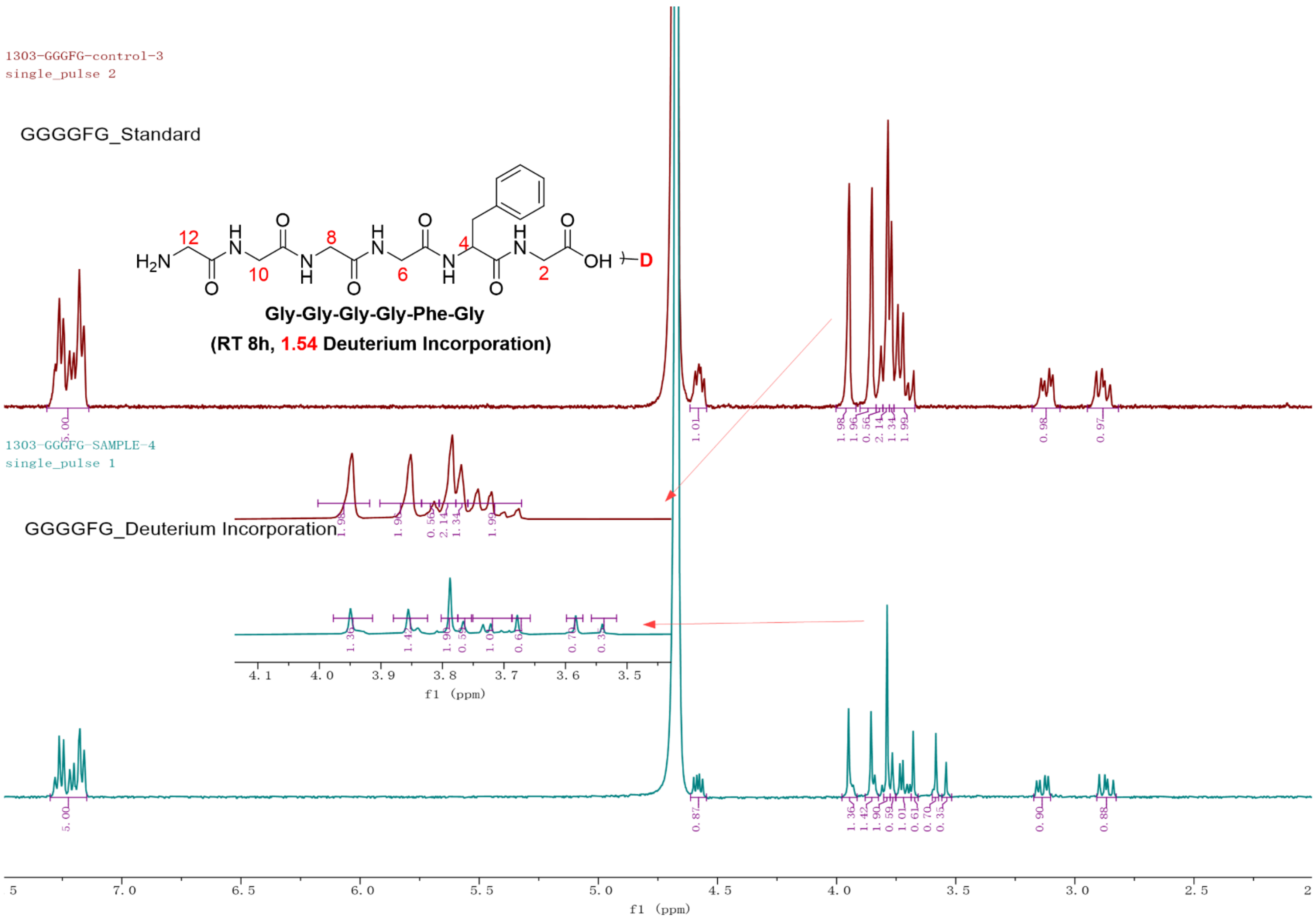
1303-GGGFG-control-3
single_pulse 2
GGGGFG_Standard
Gly-Gly-Gly-Gly-Phe-Gly
(RT 8h, 1.54 Deuterium Incorporation)
1303-GGGFG-SAMPLE-4
single_pulse 1
GGGGFG_Deuterium Incorporation
f1 (ppm)

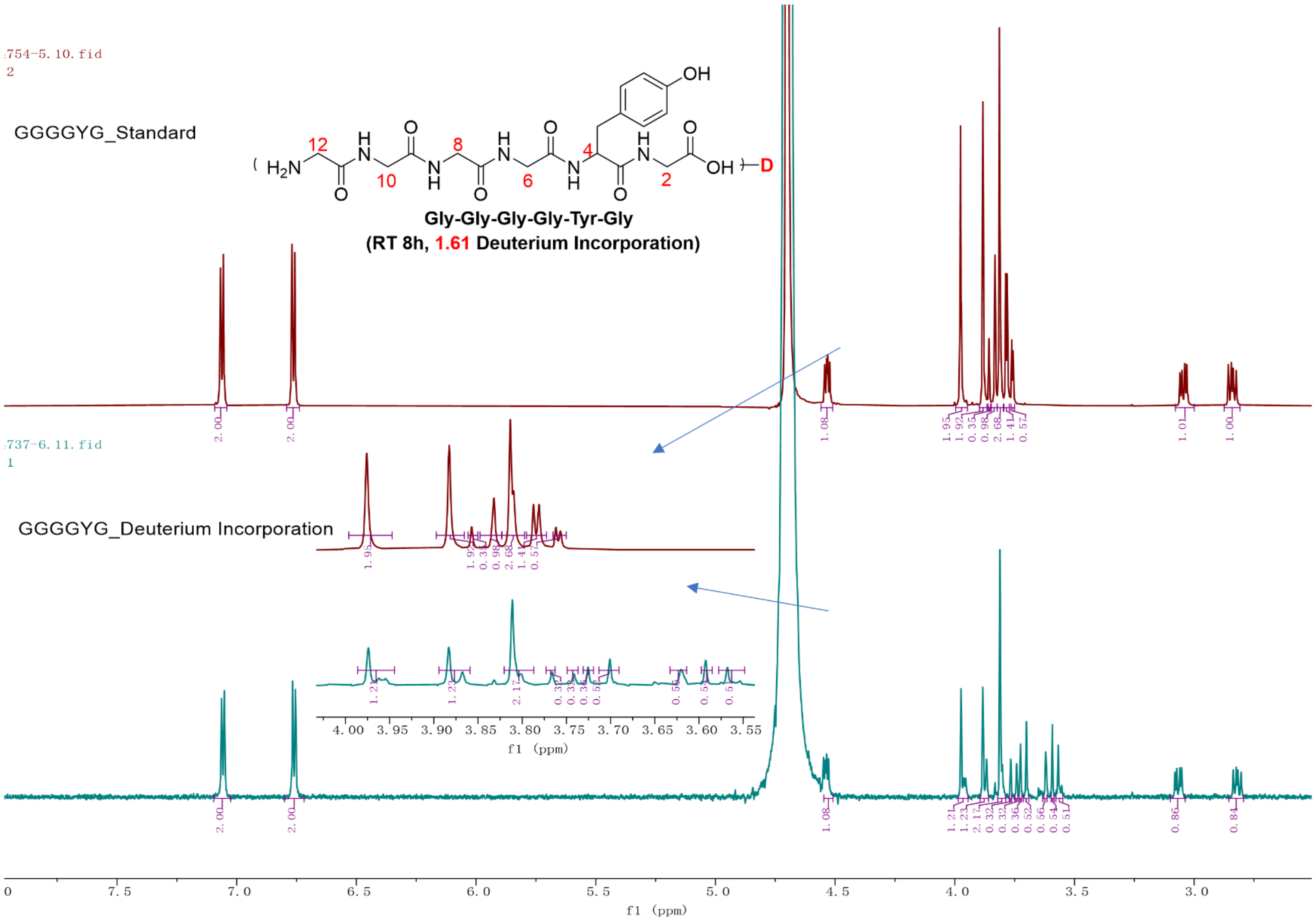

GGGGYG_Standard
GGGGYG_Deuterium Incorporation
Gly-Gly-Gly-Gly-Tyr-Gly
(RT 8h, 1.61 Deuterium Incorporation)
f1 (ppm)

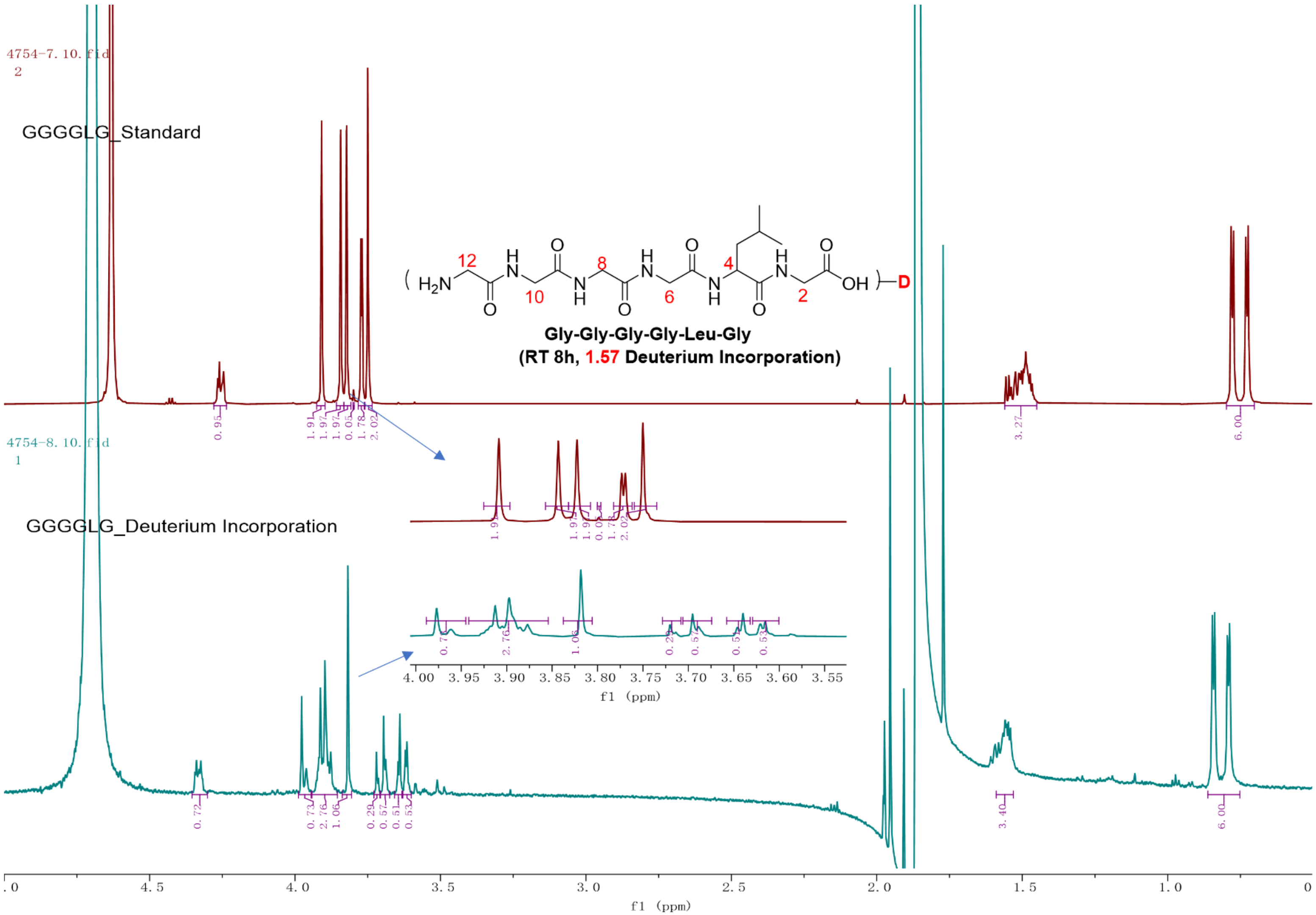

4754-7.10.fid
2
GGGGLG_Standard
4754-8.10.fid
1
GGGGLG_Deuterium Incorporation
Gly-Gly-Gly-Gly-Leu-Gly
(RT 8h, 1.57 Deuterium Incorporation)
f1 (ppm)

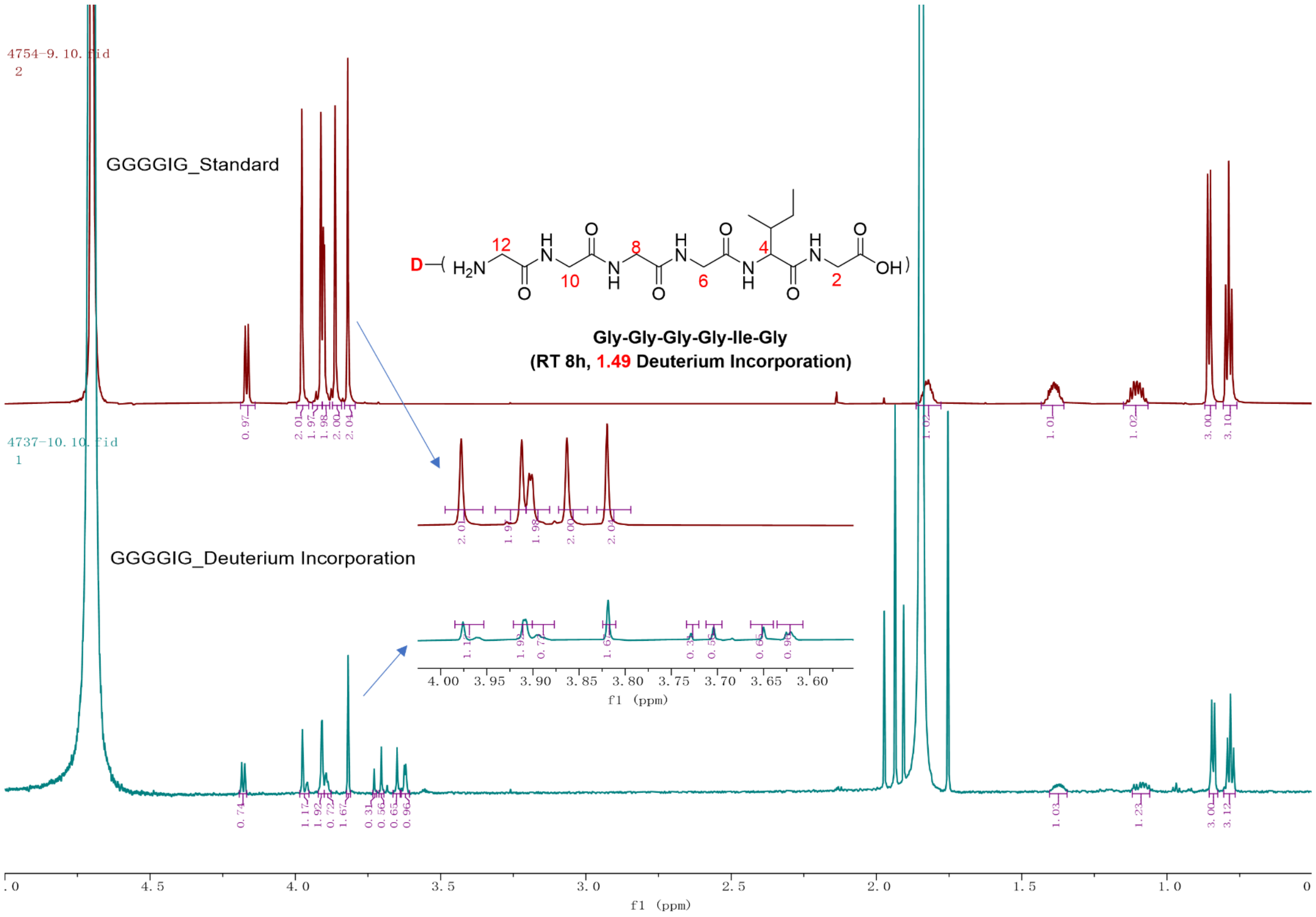

4754-9.10.fid
2
GGGGIG_Standard
4737-10.10.fid
1
GGGGIG_Deuterium Incorporation
Gly-Gly-Gly-Gly-Ile-Gly
(RT 8h, 1.49 Deuterium Incorporation)
f1 (ppm)

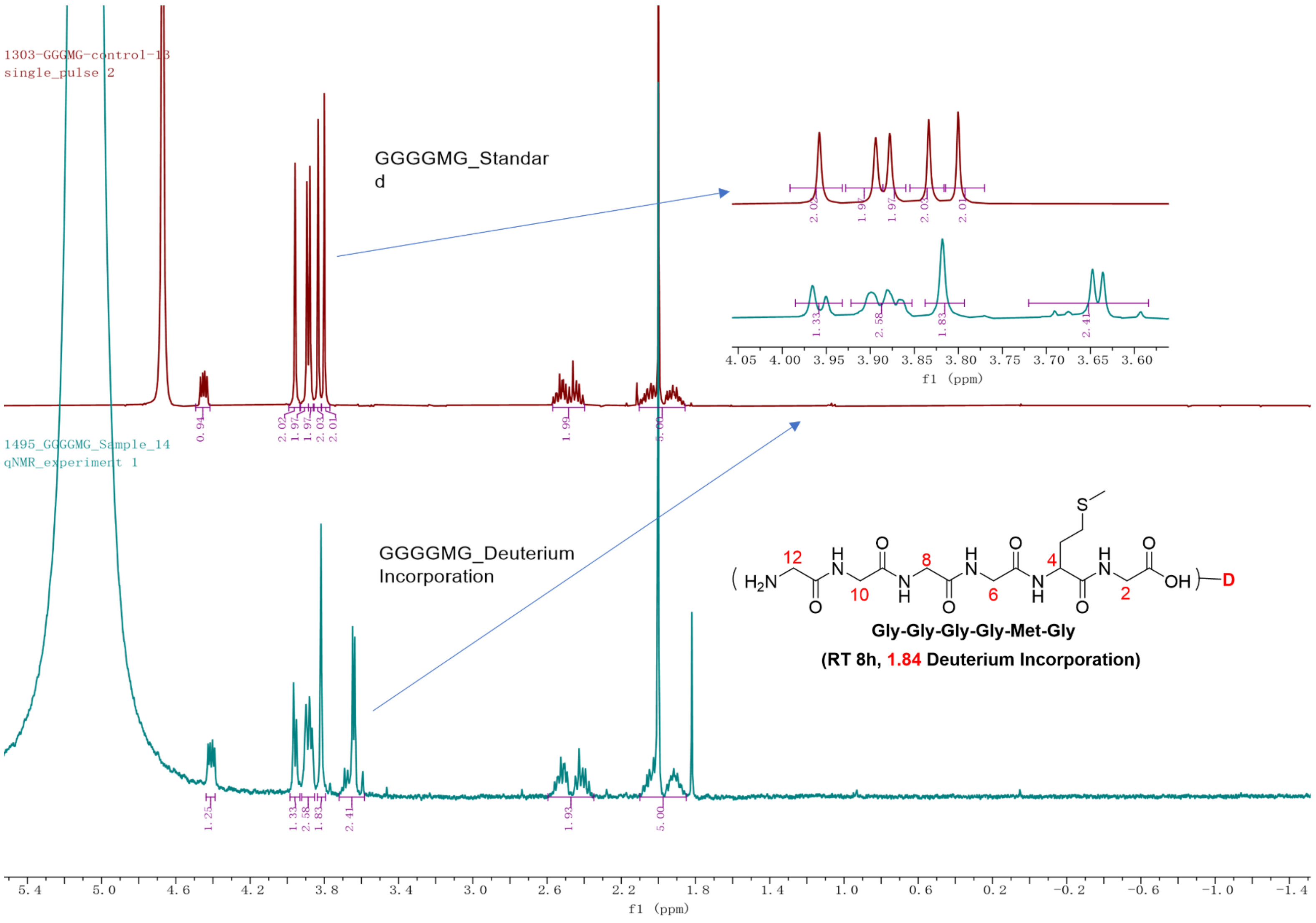
1303-GGGMG-control-13
single_pulse 2
1495_GGGMG_Sample_14
qNMR_experiment 1
GGGGMG_Standard
GGGGMG_Deuterium Incorporation
Gly-Gly-Gly-Gly-Met-Gly
(RT 8h, 1.84 Deuterium Incorporation)
f1 (ppm)

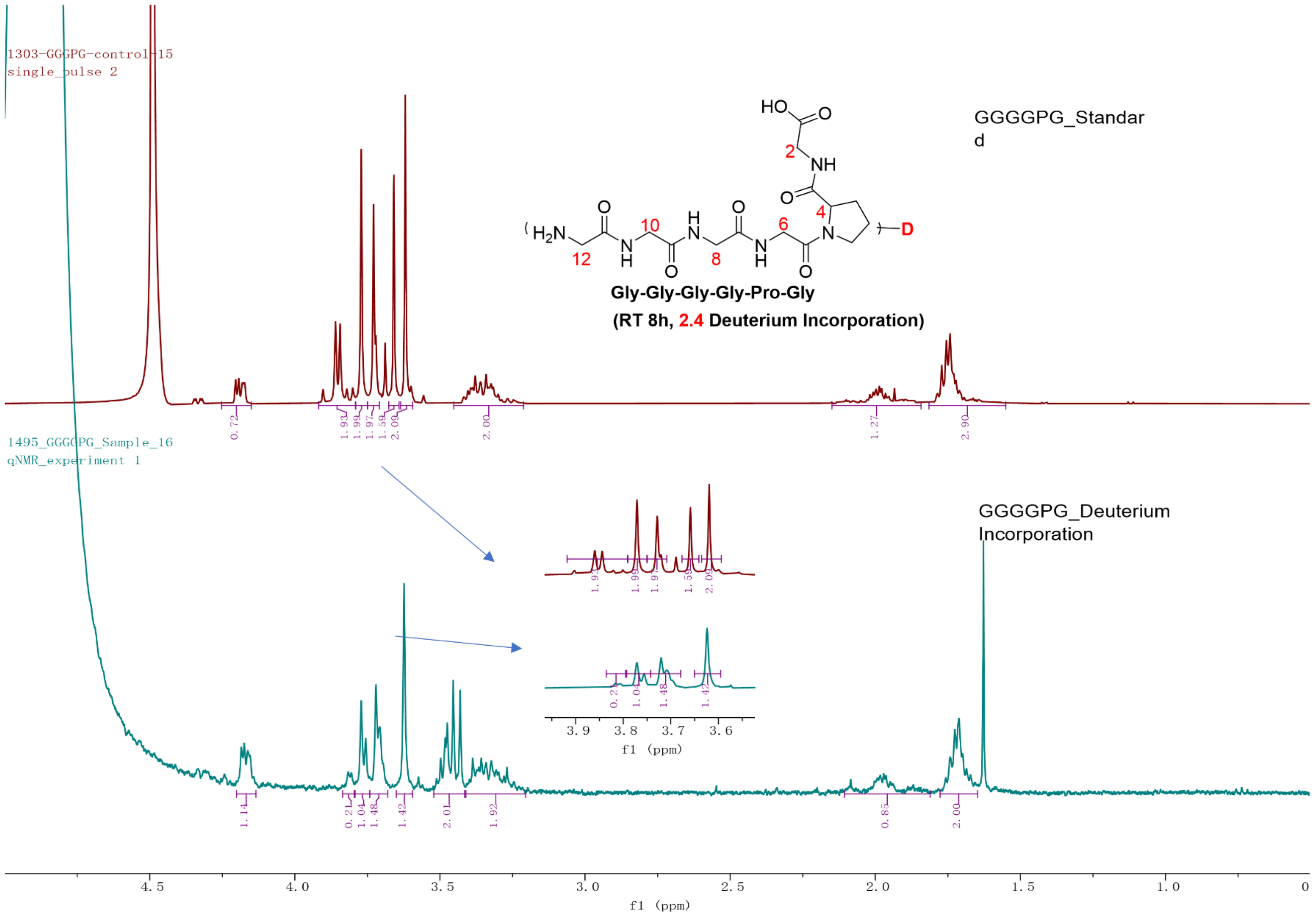
1303-GGGPG-control-15
single_pulse 2
1495_GGGGPG_Sample_16
qNMR_experiment 1
GGGGPG_Standard
GGGGPG_Deuterium Incorporation
Gly-Gly-Gly-Gly-Pro-Gly
(RT 8h, 2.4 Deuterium Incorporation)
f1 (ppm)

Part IIII. Supporting Information HPLC-MS/MS Part

Figure S6 The HPLC-MS spectra of the standard hexapeptides.

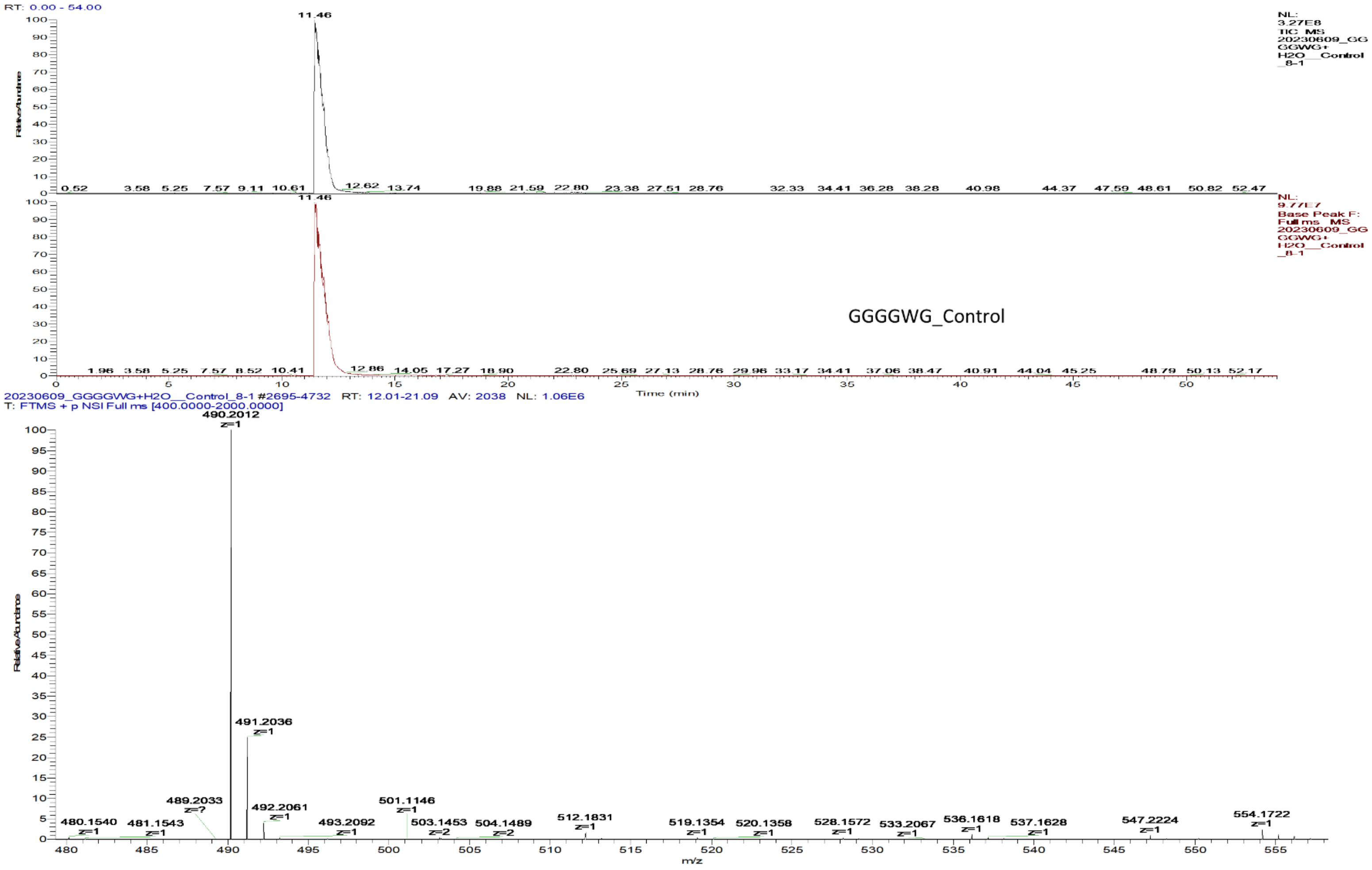
RT: 0.00 - 54.00
NL: 3.27E8 TIC MS 20230609_GGGGWG+H2O__Control_8-1
NL: 9.77E7 Base Peak F: Full ms MS 20230609_GGGGWG+H2O__Control_8-1
GGGGWG_Control
Relative Abundance
Time (min)
20230609_GGGGWG+H2O__Control_8-1 #2695-4732 RT: 12.01-21.09 AV: 2038 NL: 1.06E6
T: FTMS + p NSI Full ms [400.0000-2000.0000]
490.2012 z=1
491.2036 z=1
489.2033 z=?
492.2061 z=1
501.1146 z=1
m/z

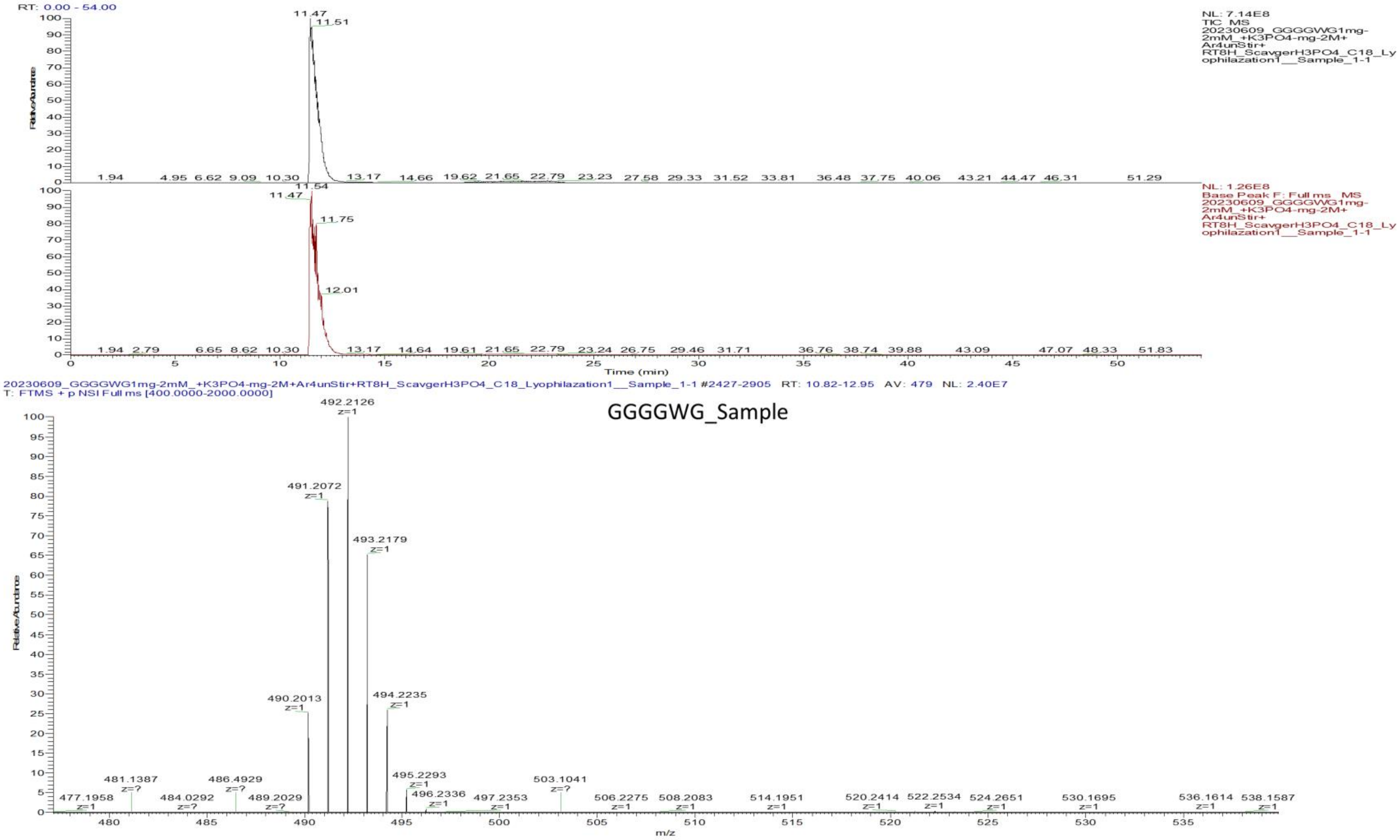
RT: 0.00 - 54.00
NL: 7.14E8
TIC MS
20230609_GGGGWG1mg-2mM_+K3PO4-mg-2M+Ar4unStir+RT8H_ScavgerH3PO4_C18_Lyophilazation1__Sample_1-1
NL: 1.26E8
Base Peak F: Full ms MS
20230609_GGGGWG1mg-2mM_+K3PO4-mg-2M+Ar4unStir+RT8H_ScavgerH3PO4_C18_Lyophilazation1__Sample_1-1
Time (min)
20230609_GGGGWG1mg-2mM_+K3PO4-mg-2M+Ar4unStir+RT8H_ScavgerH3PO4_C18_Lyophilazation1__Sample_1-1 #2427-2905 RT: 10.82-12.95 AV: 479 NL: 2.40E7
T: FTMS + p NSI Full ms [400.0000-2000.0000]
GGGGWG_Sample
492.2126 z=1
491.2072 z=1
493.2179 z=1
490.2013 z=1
494.2235 z=1
495.2293 z=1
496.2336 z=1
503.1041 z=?
481.1387 z=?
486.4929 z=?
Relative Abundance
m/z

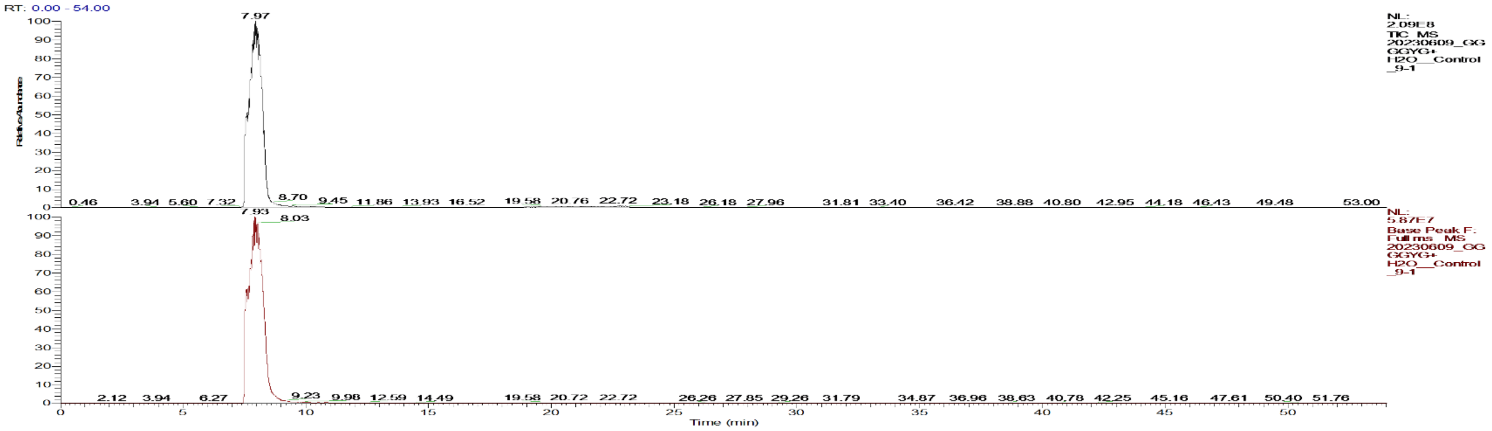

RT: 0.00 - 54.00
NL: 2.09E8 TIC MS 20230609_GGGGYG+H2O__Control_9-1
NL: 5.87E7 Base Peak F: Full ms MS 20230609_GGGGYG+H2O__Control_9-1
Relative Abundance
Time (min)

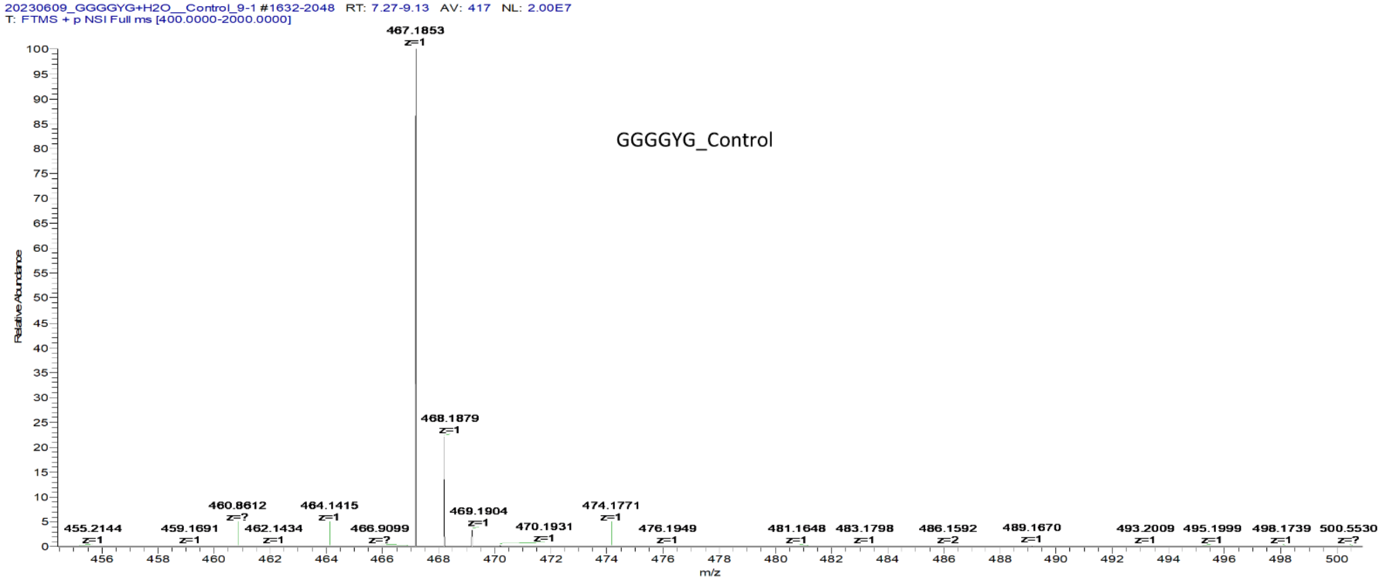

20230609_GGGGYG+H2O__Control_9-1 #1632-2048 RT: 7.27-9.13 AV: 417 NL: 2.00E7
T: FTMS + p NSI Full ms [400.0000-2000.0000]
467.1853 z=1
468.1879 z=1
GGGGYG_Control
Relative Abundance
m/z

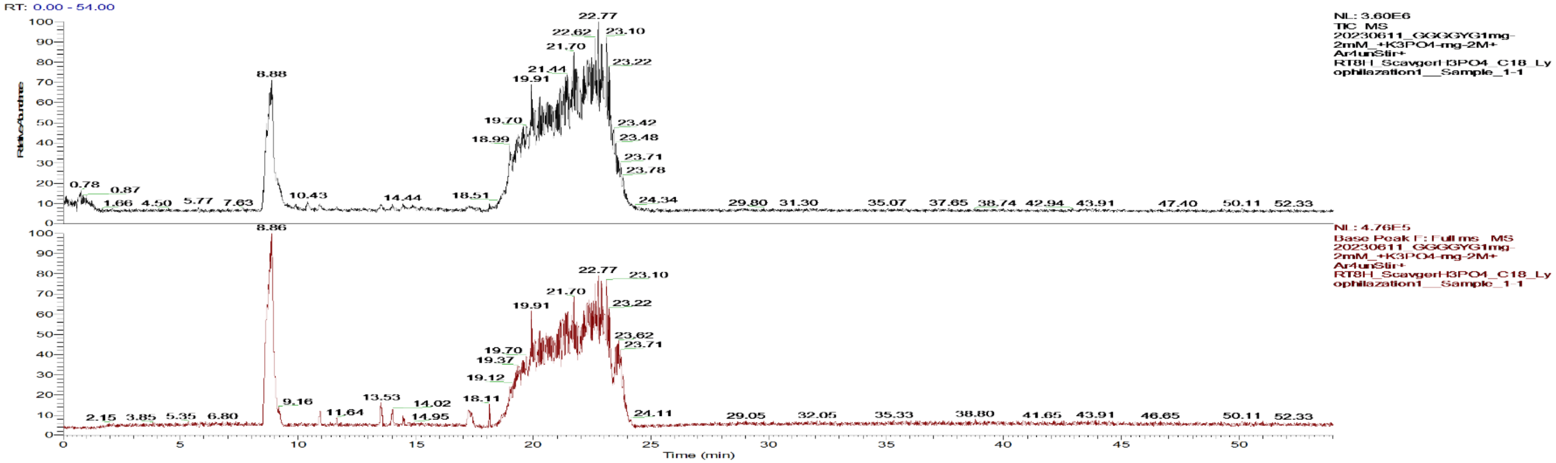
RT: 0.00 - 54.00
NL: 3.60E6
TIC MS
20230611_GGGGYG1mg-2mM_+K3PO4-mg-2M+Ar4unStir+RT8H_ScavgerH3PO4_C18_Lyophilazation1__Sample_1-1
NL: 4.76E5
Base Peak F: Full ms MS
20230611_GGGGYG1mg-2mM_+K3PO4-mg-2M+Ar4unStir+RT8H_ScavgerH3PO4_C18_Lyophilazation1__Sample_1-1
Relative Abundance
Time (min)

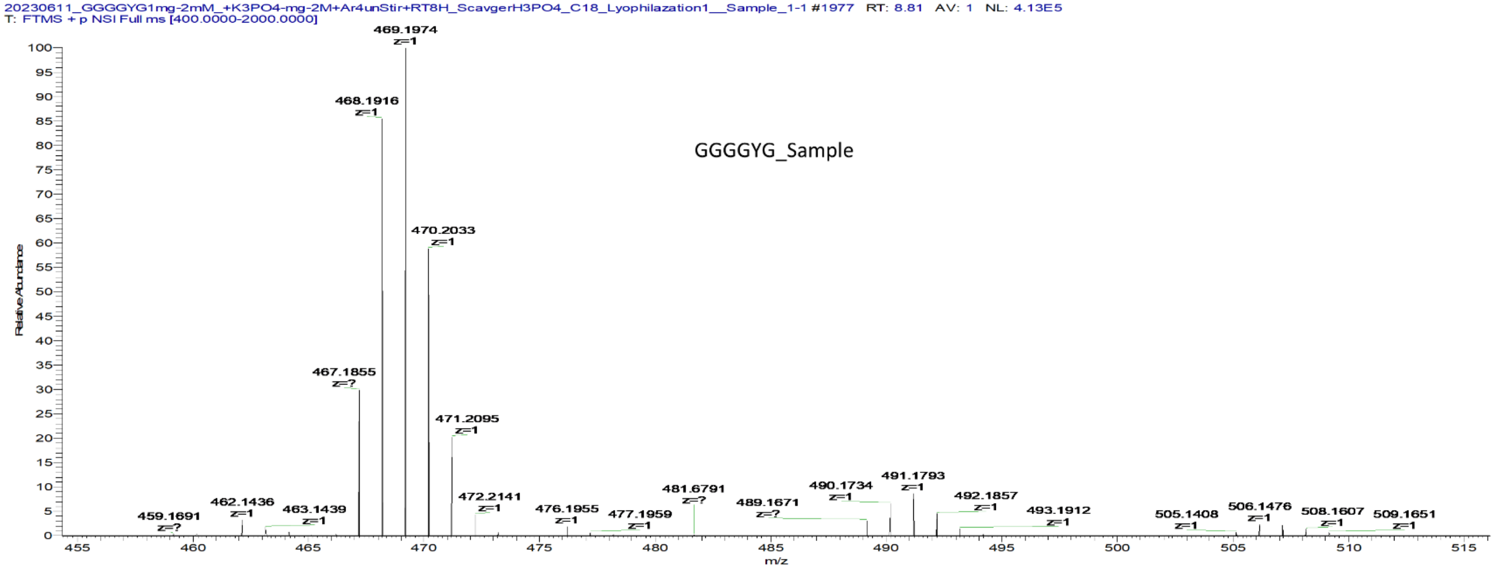
20230611_GGGGYG1mg-2mM_+K3PO4-mg-2M+Ar4unStir+RT8H_ScavgerH3PO4_C18_Lyophilazation1__Sample_1-1 #1977 RT: 8.81 AV: 1 NL: 4.13E5
T: FTMS + p NSI Full ms [400.0000-2000.0000]
GGGGYG_Sample
469.1974 z=1
468.1916 z=1
470.2033 z=1
467.1855 z=?
471.2095 z=1
Relative Abundance
m/z

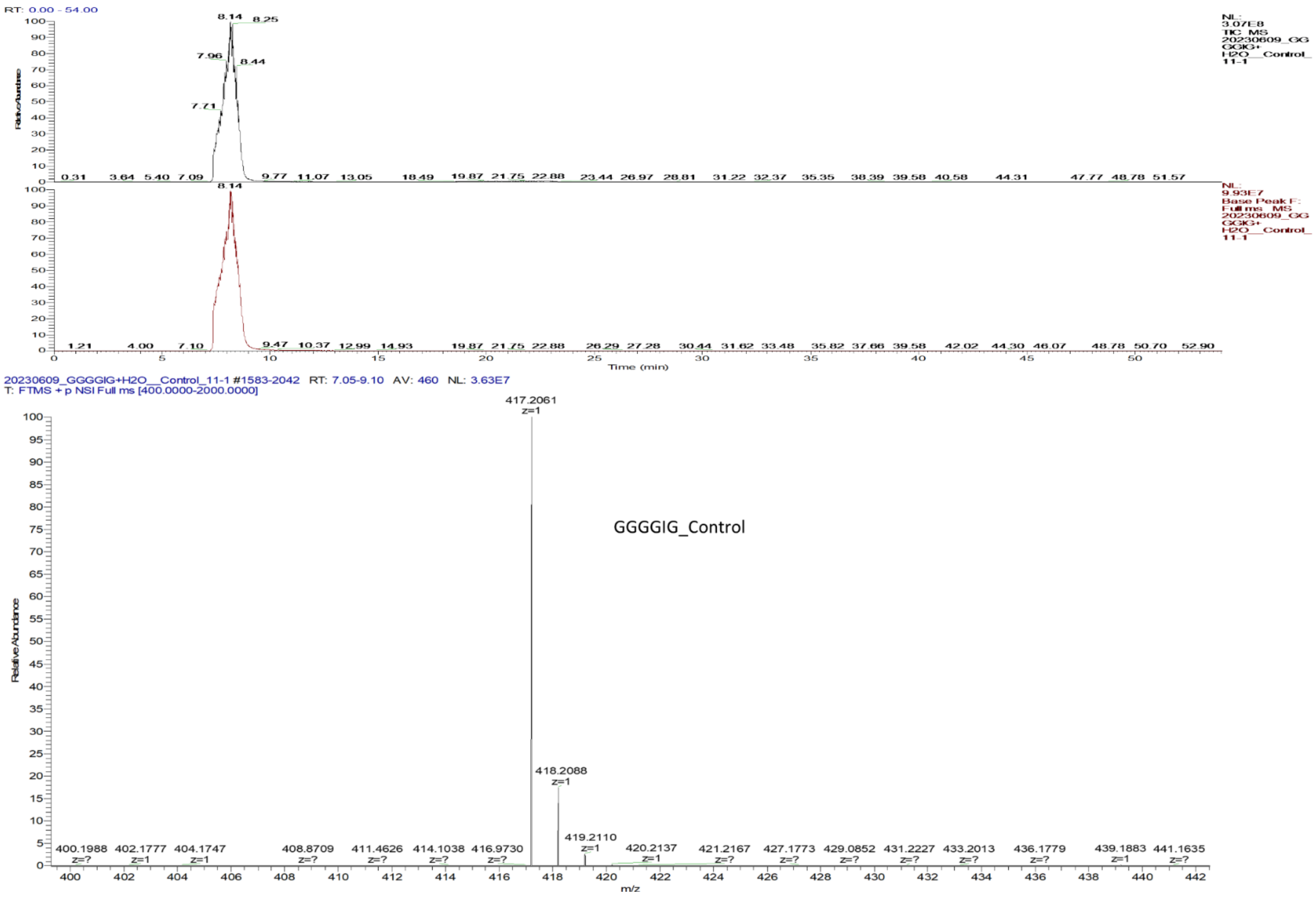

RT: 0.00 - 54.00
NL: 3.07E8 TIC MS 20230609_GGGIG+H2O__Control_11-1
NL: 9.93E7 Base Peak F: Full ms MS 20230609_GGGIG+H2O__Control_11-1
Relative Abundance
Time (min)
20230609_GGGGIG+H2O__Control_11-1 #1583-2042 RT: 7.05-9.10 AV: 460 NL: 3.63E7
T: FTMS + p NSI Full ms [400.0000-2000.0000]
417.2061 z=1
418.2088 z=1
419.2110 z=1
GGGGIG_Control
m/z

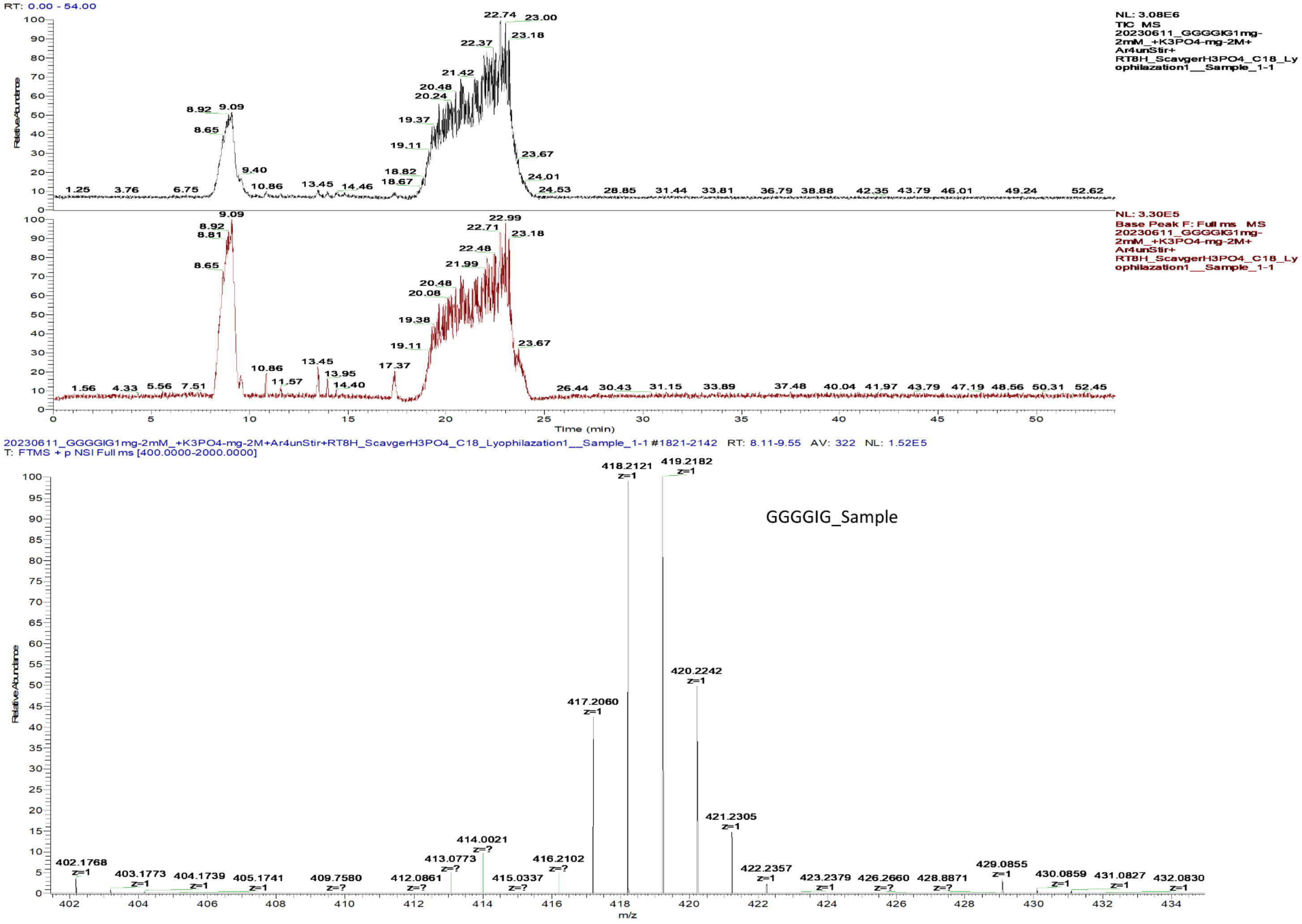
RT: 0.00 - 54.00
NL: 3.08E6
TIC MS
20230611_GGGGIG1mg-2mM_+K3PO4-mg-2M+Ar4unStir+RT8H_ScavgerH3PO4_C18_Lyophilazation1__Sample_1-1
NL: 3.30E5
Base Peak F: Full ms MS
20230611_GGGGIG1mg-2mM_+K3PO4-mg-2M+Ar4unStir+RT8H_ScavgerH3PO4_C18_Lyophilazation1__Sample_1-1
Relative Abundance
Time (min)
20230611_GGGGIG1mg-2mM_+K3PO4-mg-2M+Ar4unStir+RT8H_ScavgerH3PO4_C18_Lyophilazation1__Sample_1-1 #1821-2142 RT: 8.11-9.55 AV: 322 NL: 1.52E5
T: FTMS + p NSI Full ms [400.0000-2000.0000]
GGGGIG_Sample
418.2121 z=1
419.2182 z=1
417.2060 z=1
420.2242 z=1
421.2305 z=1
414.0021 z=?
m/z

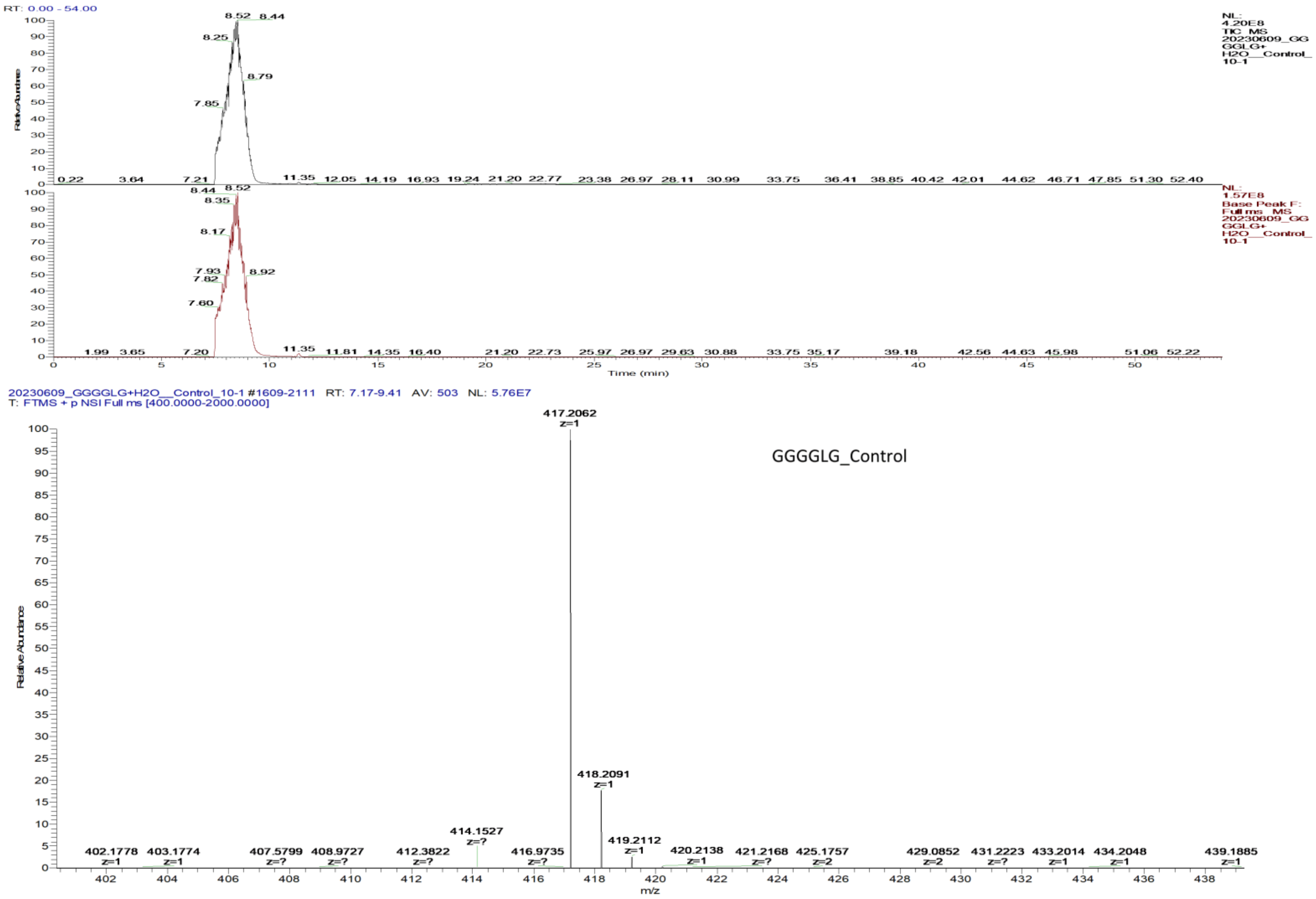
RT: 0.00 - 54.00
NL: 4.20E8 TIC MS 20230609_GGGGLG+H2O__Control_10-1
NL: 1.57E8 Base Peak F: Full ms MS 20230609_GGGGLG+H2O__Control_10-1
Time (min)
Relative Abundance
20230609_GGGGLG+H2O__Control_10-1 #1609-2111 RT: 7.17-9.41 AV: 503 NL: 5.76E7
T: FTMS + p NSI Full ms [400.0000-2000.0000]
417.2062 z=1
418.2091 z=1
419.2112 z=1
414.1527 z=?
GGGGLG_Control
m/z

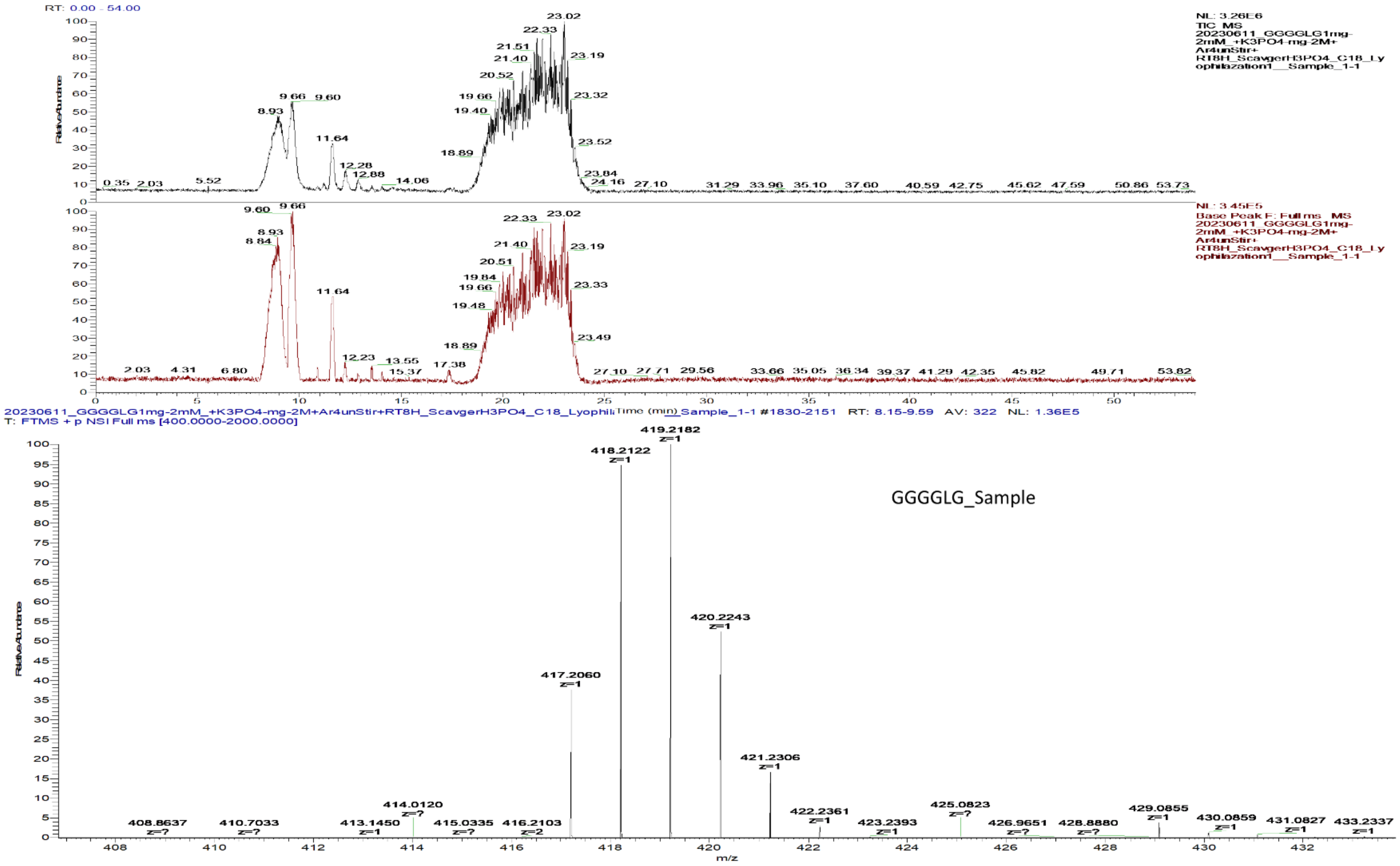
RT: 0.00 - 54.00
NL: 3.26E6
TIC MS
20230611_GGGGLG1mg-2mM_+K3PO4-mg-2M+Ar4unStir+RT8H_ScavgerH3PO4_C18_Lyophilazation1__Sample_1-1
NL: 3.45E5
Base Peak F: Full ms MS
20230611_GGGGLG1mg-2mM_+K3PO4-mg-2M+Ar4unStir+RT8H_ScavgerH3PO4_C18_Lyophilazation1__Sample_1-1
Time (min)
20230611_GGGGLG1mg-2mM_+K3PO4-mg-2M+Ar4unStir+RT8H_ScavgerH3PO4_C18_Lyophil... #1830-2151 RT: 8.15-9.59 AV: 322 NL: 1.36E5
T: FTMS + p NSI Full ms [400.0000-2000.0000]
GGGGLG_Sample
419.2182 z=1
418.2122 z=1
420.2243 z=1
417.2060 z=1
421.2306 z=1
m/z

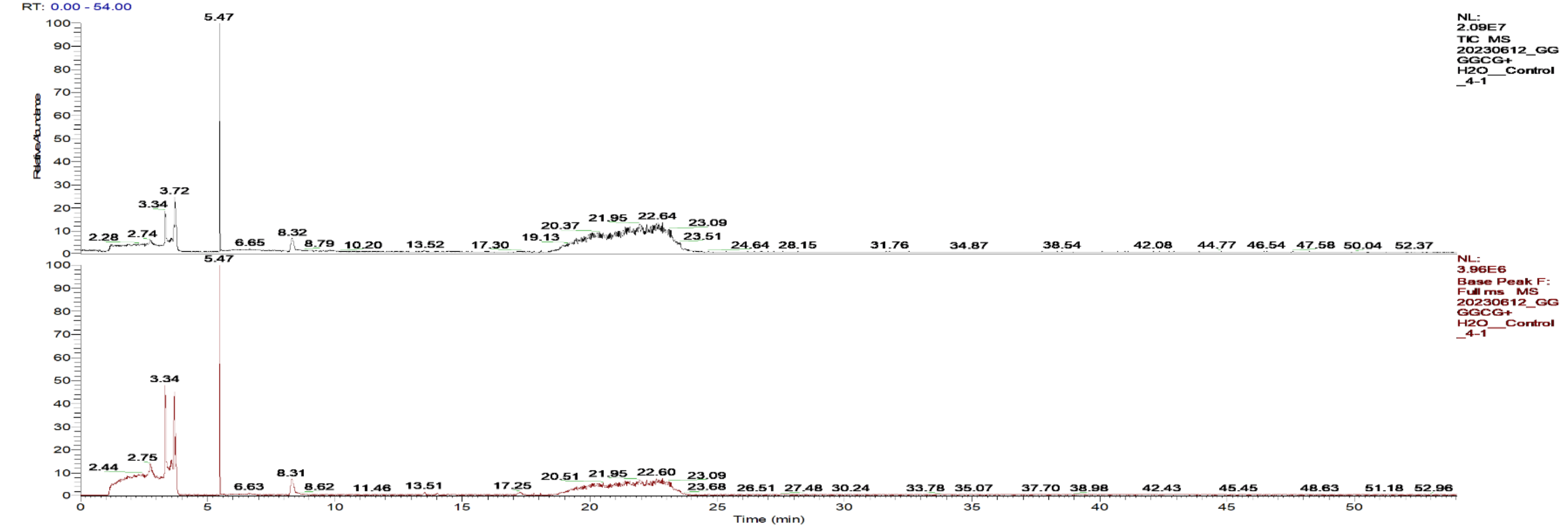
RT: 0.00 - 54.00
NL: 2.09E7 TIC MS 20230612_GGGGCG+H2O__Control_4-1
NL: 3.96E6 Base Peak F: Full ms MS 20230612_GGGGCG+H2O__Control_4-1
Time (min)
Relative Abundance

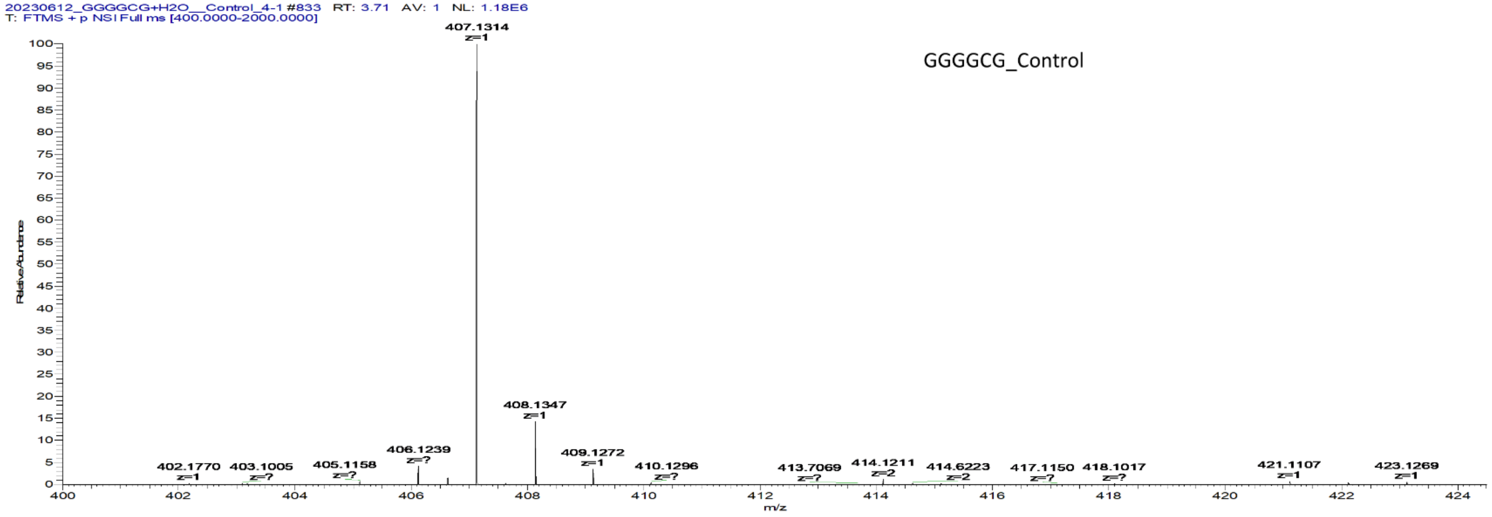
20230612_GGGGCG+H2O__Control_4-1 #833 RT: 3.71 AV: 1 NL: 1.18E6
T: FTMS + p NSI Full ms [400.0000-2000.0000]
GGGGCG_Control
407.1314
408.1347
409.1272
406.1239
m/z
Relative Abundance

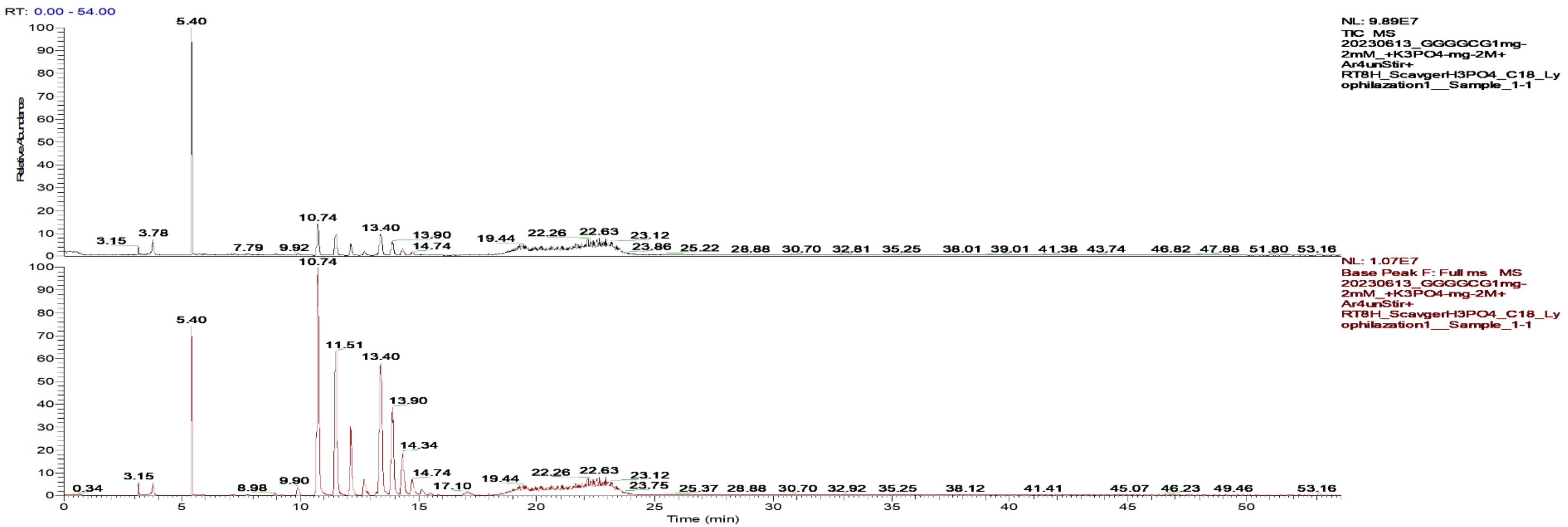
RT: 0.00 - 54.00
NL: 9.89E7
TIC MS
20230613_GGGGCG1mg-2mM_+K3PO4-mg-2M+Ar4unStir+RT8H_ScavgerH3PO4_C18_Lyophilazation1__Sample_1-1
NL: 1.07E7
Base Peak F: Full ms MS
20230613_GGGGCG1mg-2mM_+K3PO4-mg-2M+Ar4unStir+RT8H_ScavgerH3PO4_C18_Lyophilazation1__Sample_1-1
Relative Abundance
Time (min)

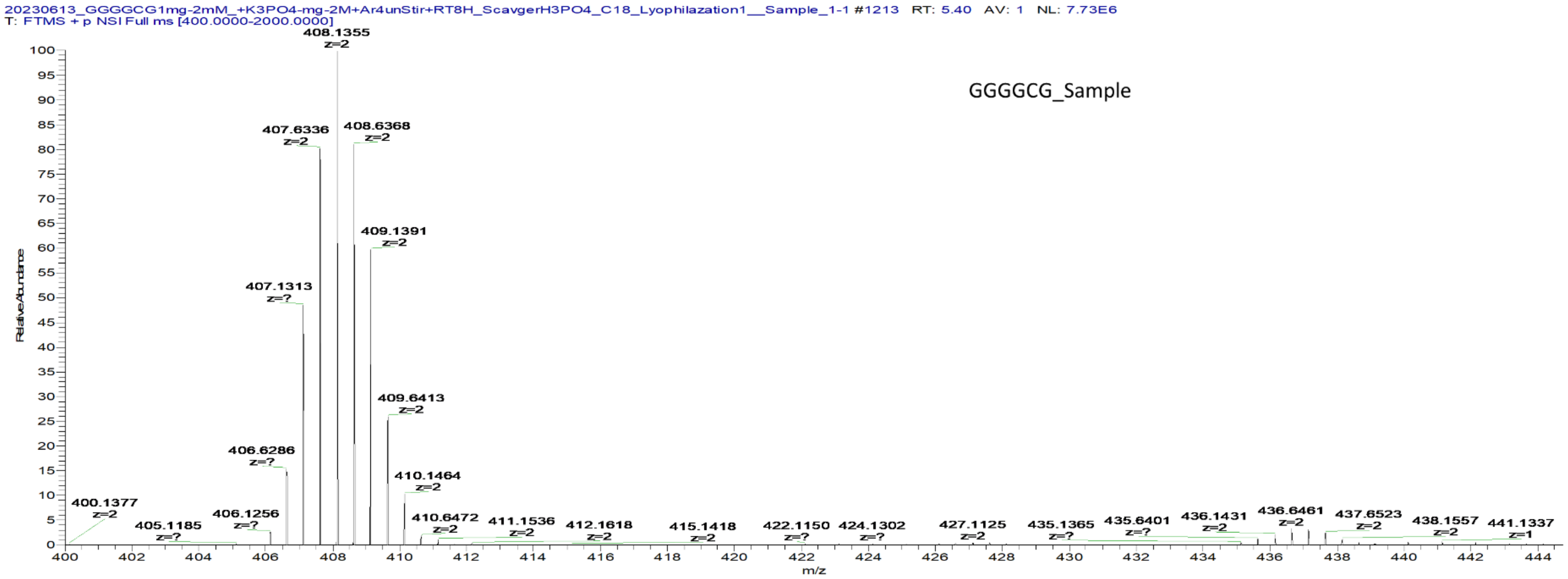
20230613_GGGGCG1mg-2mM_+K3PO4-mg-2M+Ar4unStir+RT8H_ScavgerH3PO4_C18_Lyophilazation1__Sample_1-1 #1213 RT: 5.40 AV: 1 NL: 7.73E6
T: FTMS + p NSI Full ms [400.0000-2000.0000]
GGGGCG_Sample
408.1355
407.6336
408.6368
409.1391
407.1313
409.6413
406.6286
410.1464
Relative Abundance
m/z

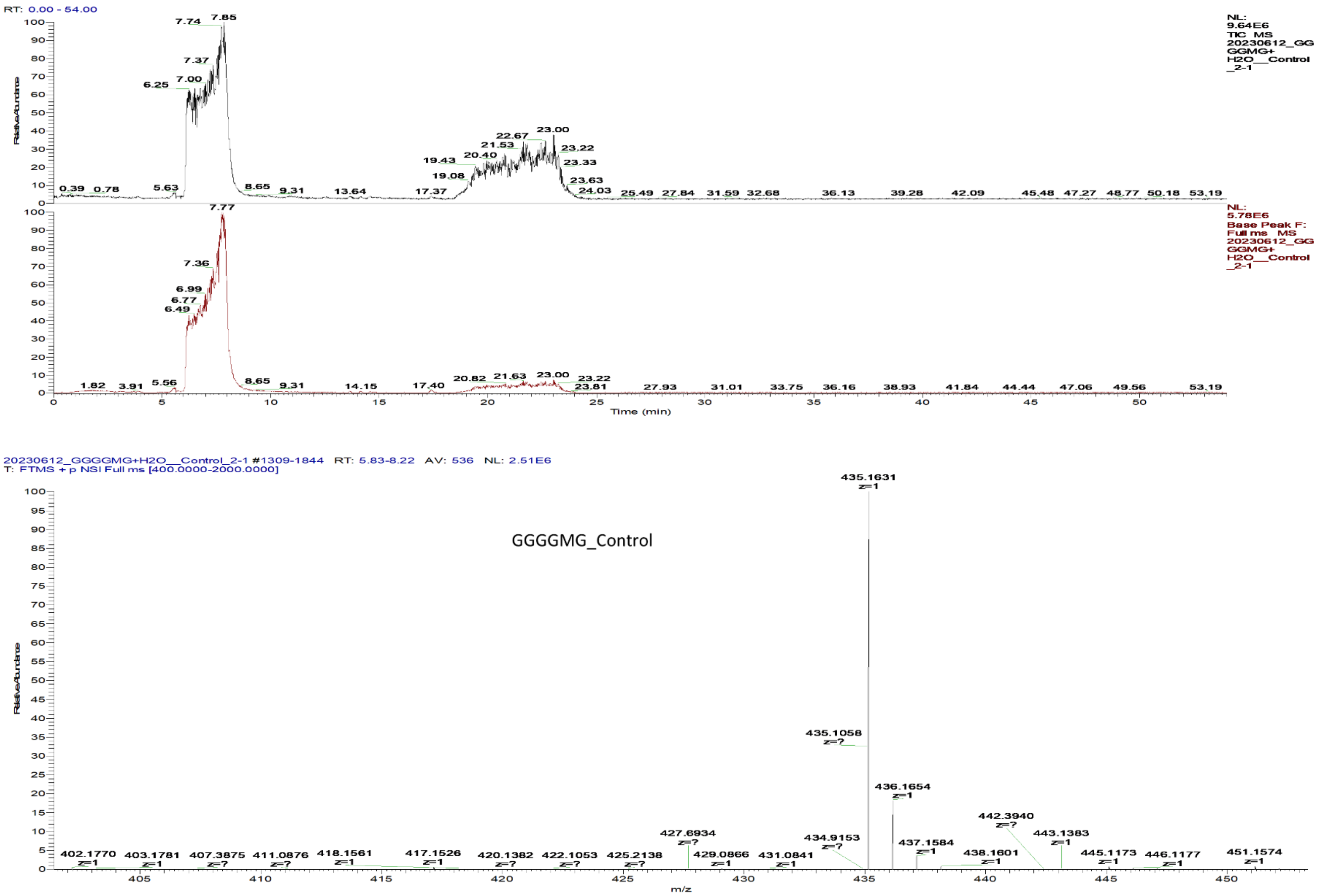
RT: 0.00 - 54.00
NL: 9.64E6 TIC MS 20230612_GGGMG+H2O__Control_2-1
NL: 5.78E6 Base Peak F: Full ms MS 20230612_GGGMG+H2O__Control_2-1
Time (min)
Relative Abundance
20230612_GGGGMG+H2O__Control_2-1 #1309-1844 RT: 5.83-8.22 AV: 536 NL: 2.51E6
T: FTMS + p NSI Full ms [400.0000-2000.0000]
GGGGMG_Control
435.1631
435.1058
436.1654
442.3940
443.1383
427.6934
434.9153
437.1584
438.1601
m/z

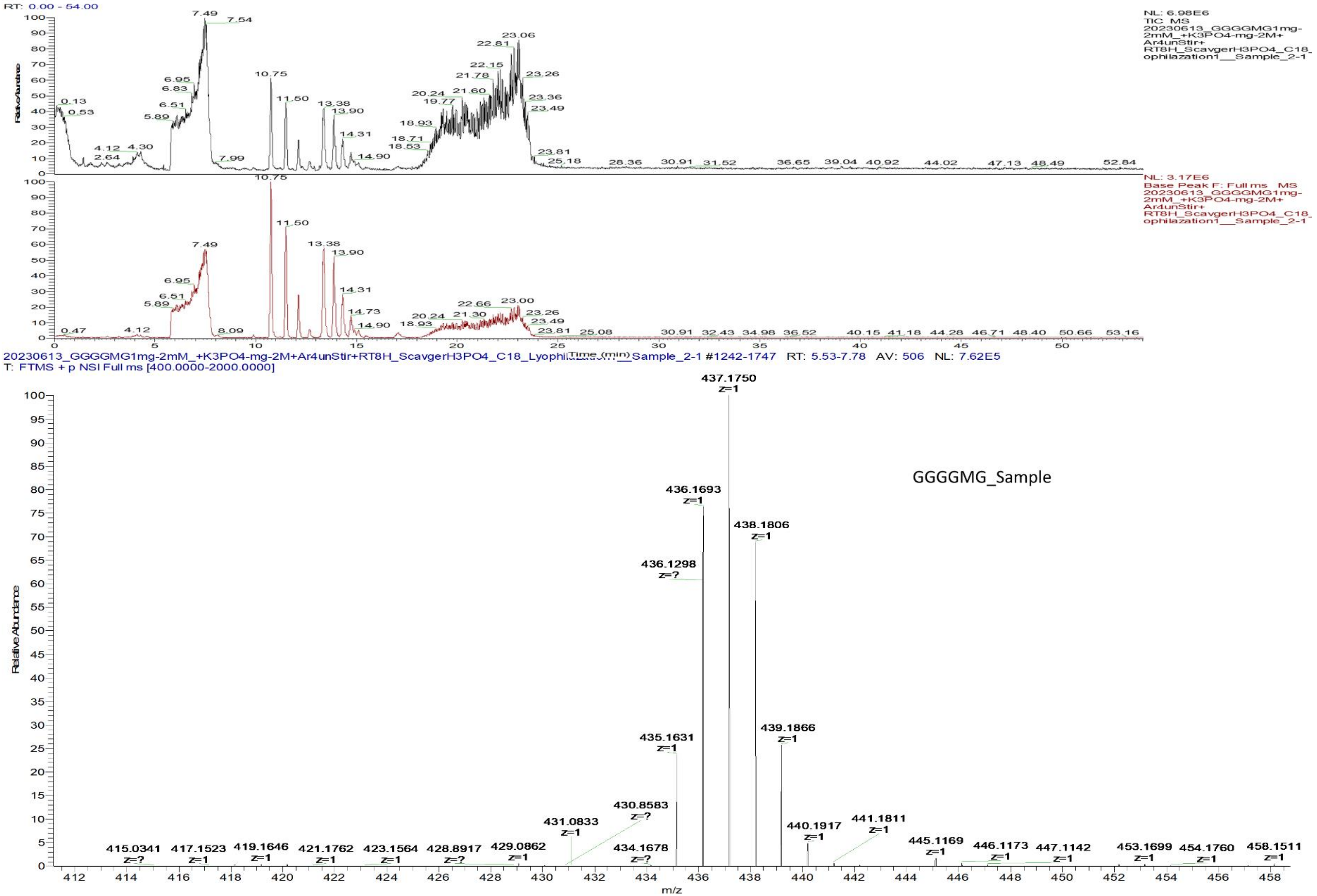
RT: 0.00 - 54.00
NL: 6.98E6
TIC MS
20230613_GGGGMG1mg-2mM_+K3PO4-mg-2M+Ar4unStir+RT8H_ScavgerH3PO4_C18_ophilazation1__Sample_2-1
NL: 3.17E6
Base Peak F: Full ms MS
20230613_GGGGMG1mg-2mM_+K3PO4-mg-2M+Ar4unStir+RT8H_ScavgerH3PO4_C18_ophilazation1__Sample_2-1
Time (min)
Relative Abundance
20230613_GGGGMG1mg-2mM_+K3PO4-mg-2M+Ar4unStir+RT8H_ScavgerH3PO4_C18_Lyophi... Sample_2-1 #1242-1747 RT: 5.53-7.78 AV: 506 NL: 7.62E5
T: FTMS + p NSI Full ms [400.0000-2000.0000]
GGGGMG_Sample
437.1750 z=1
436.1693 z=1
438.1806 z=1
436.1298 z=?
439.1866 z=1
435.1631 z=1
430.8583 z=?
431.0833 z=1
441.1811 z=1
440.1917 z=1
m/z

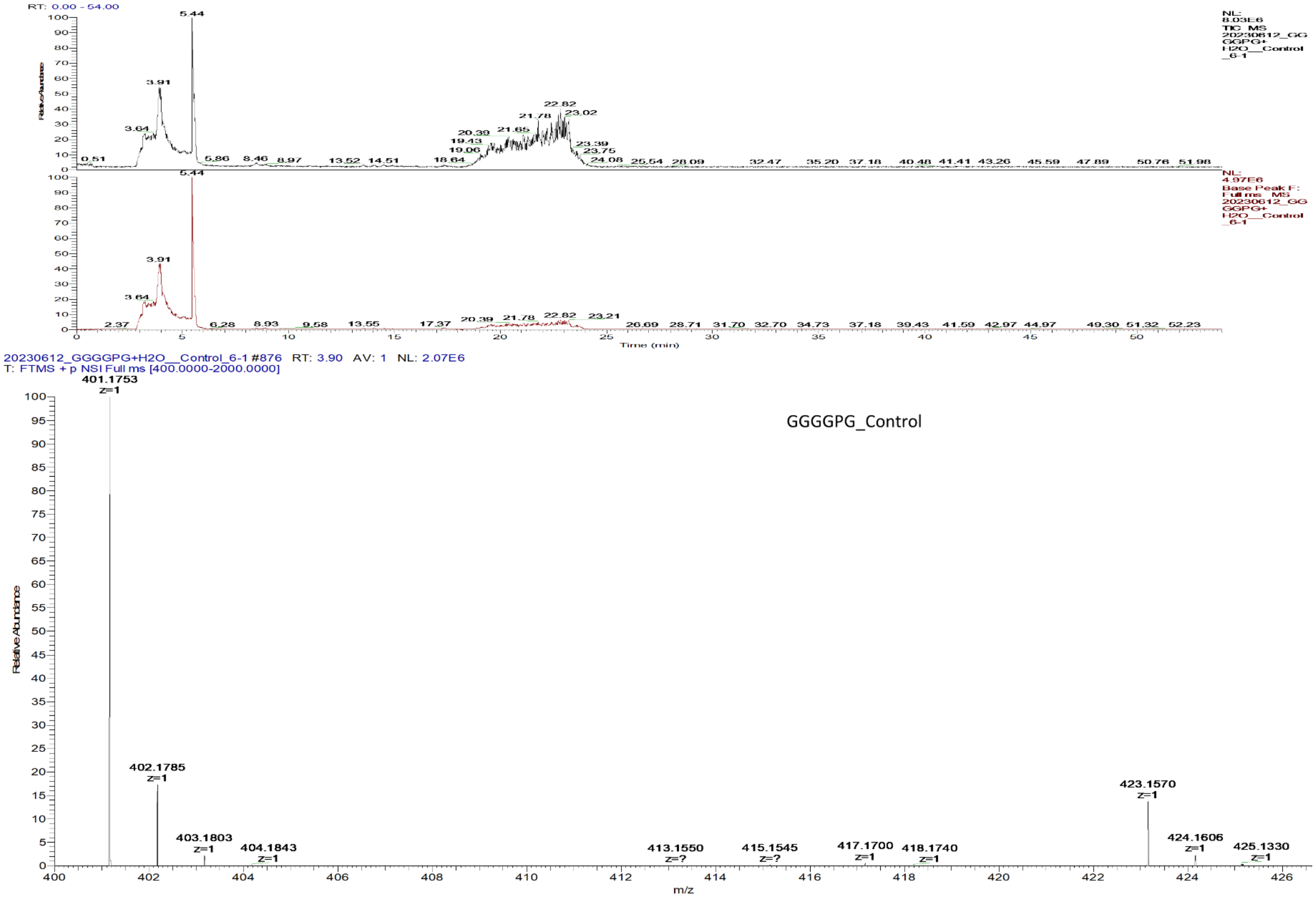
RT: 0.00 - 54.00
NL: 8.03E6 TIC MS 20230612_GGGGPG+H2O__Control_6-1
NL: 4.97E6 Base Peak F: Full ms MS 20230612_GGGGPG+H2O__Control_6-1
Time (min)
20230612_GGGGPG+H2O__Control_6-1 #876 RT: 3.90 AV: 1 NL: 2.07E6
T: FTMS + p NSI Full ms [400.0000-2000.0000]
GGGGPG_Control
Relative Abundance
m/z
401.1753 z=1
402.1785 z=1
403.1803 z=1
404.1843 z=1
413.1550 z=?
415.1545 z=?
417.1700 z=1
418.1740 z=1
423.1570 z=1
424.1606 z=1
425.1330 z=1

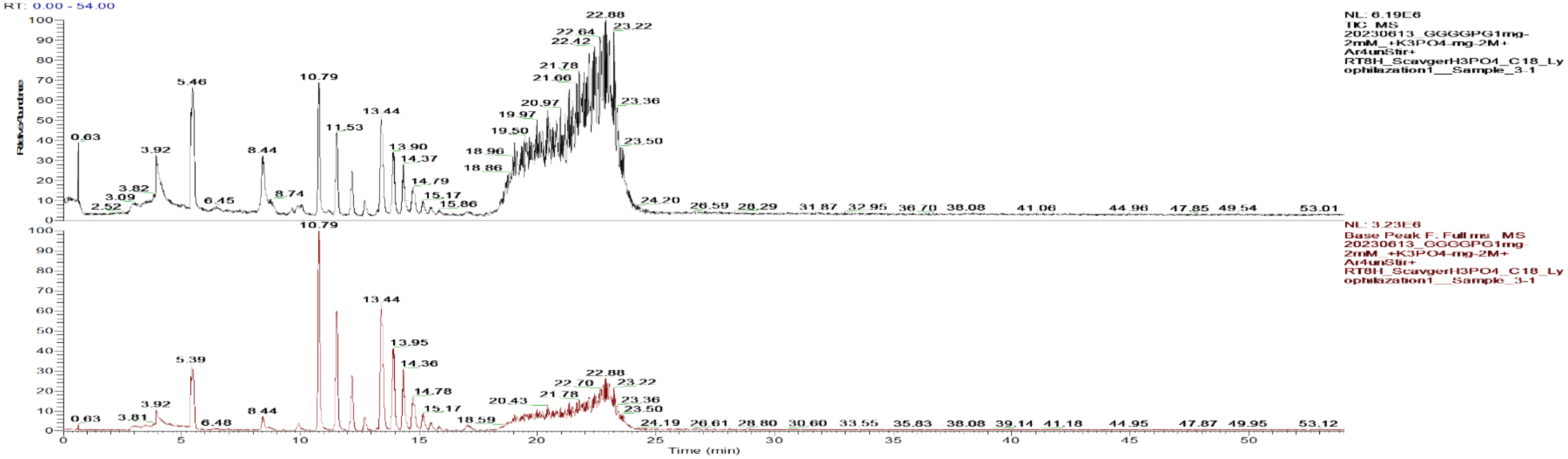
RT: 0.00 - 54.00
NL: 6.19E6
TIC MS
20230613_GGGGPG1mg-2mM_+K3PO4-mg-2M+Ar4unStir+RT8H_ScavgerH3PO4_C18_Lyophilazation1__Sample_3-1
NL: 3.23E6
Base Peak F: Full ms MS
20230613_GGGGPG1mg 2mM_+K3PO4-mg-2M+Ar4unStir+RT8H_ScavgerH3PO4_C18_Lyophilazation1__Sample_3-1
Relative Abundance
Time (min)

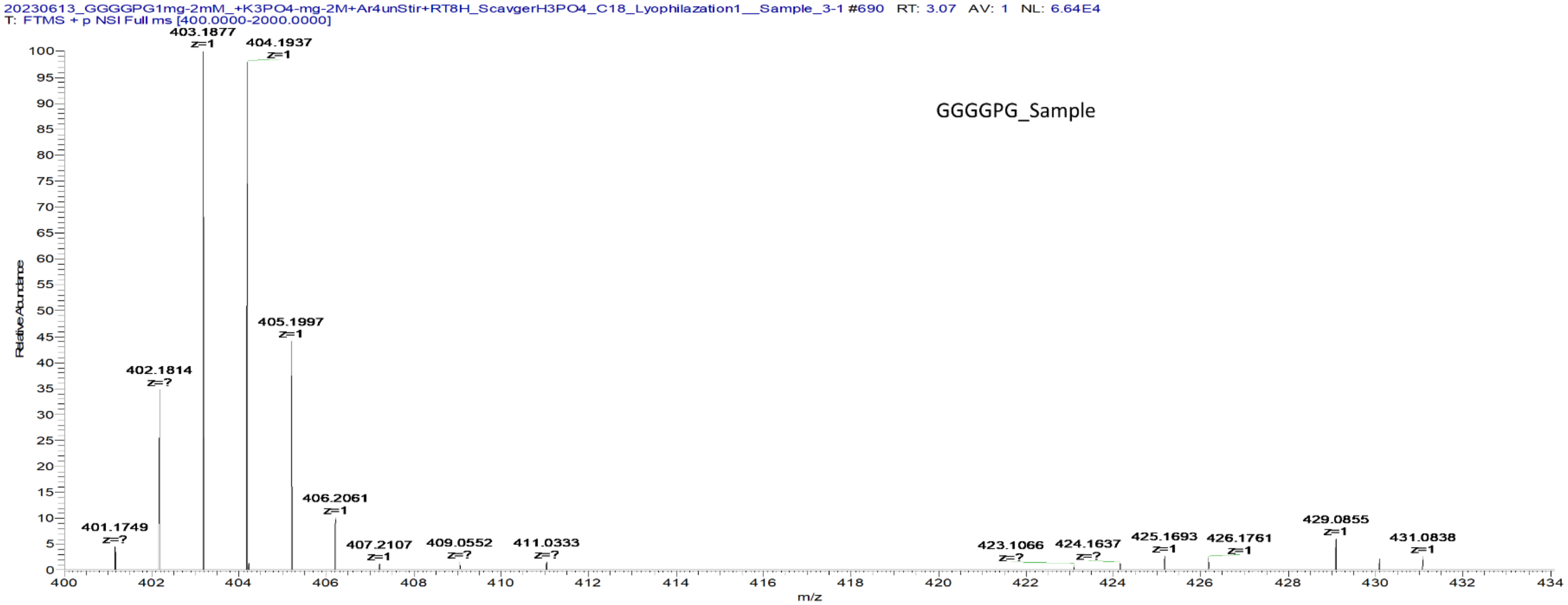
20230613_GGGGPG1mg-2mM_+K3PO4-mg-2M+Ar4unStir+RT8H_ScavgerH3PO4_C18_Lyophilazation1__Sample_3-1 #690 RT: 3.07 AV: 1 NL: 6.64E4
T: FTMS + p NSI Full ms [400.0000-2000.0000]
GGGGPG_Sample
Relative Abundance
m/z

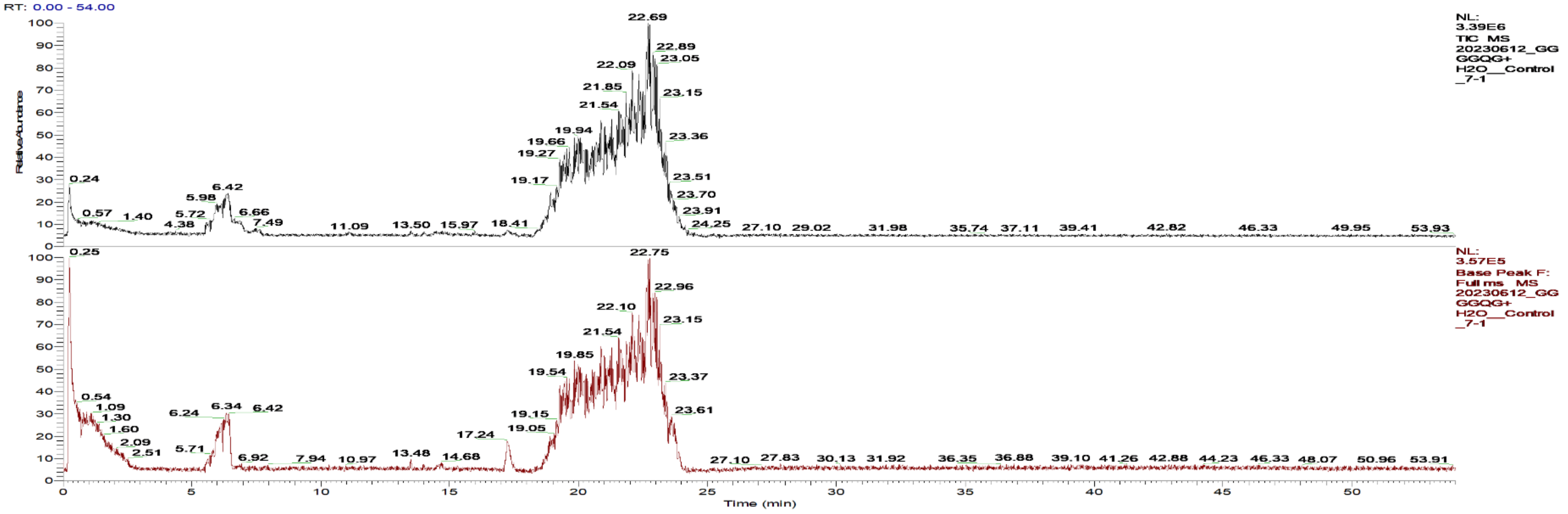
RT: 0.00 - 54.00
NL: 3.39E6 TIC MS 20230612_GGGGQG+H2O__Control_7-1
NL: 3.57E5 Base Peak F: Full ms MS 20230612_GGGGQG+H2O__Control_7-1
Relative Abundance
Time (min)

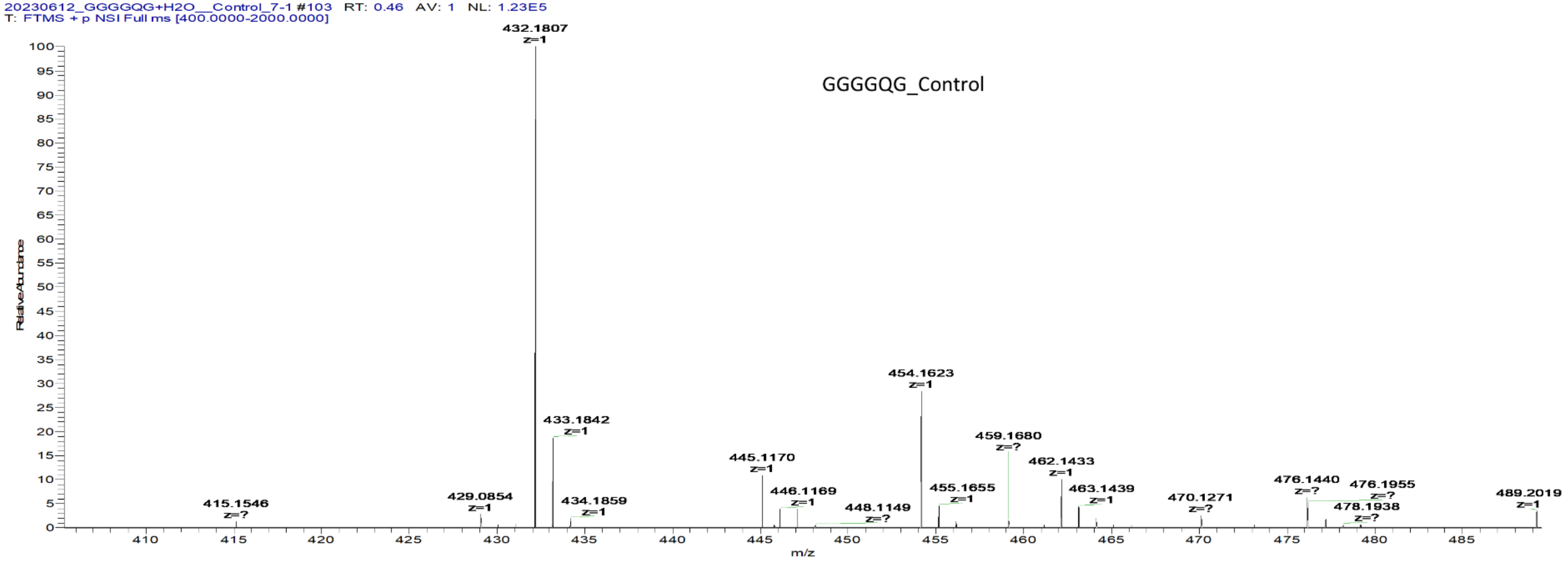
20230612_GGGGQG+H2O__Control_7-1 #103 RT: 0.46 AV: 1 NL: 1.23E5
T: FTMS + p NSI Full ms [400.0000-2000.0000]
GGGGQG_Control
Relative Abundance
m/z

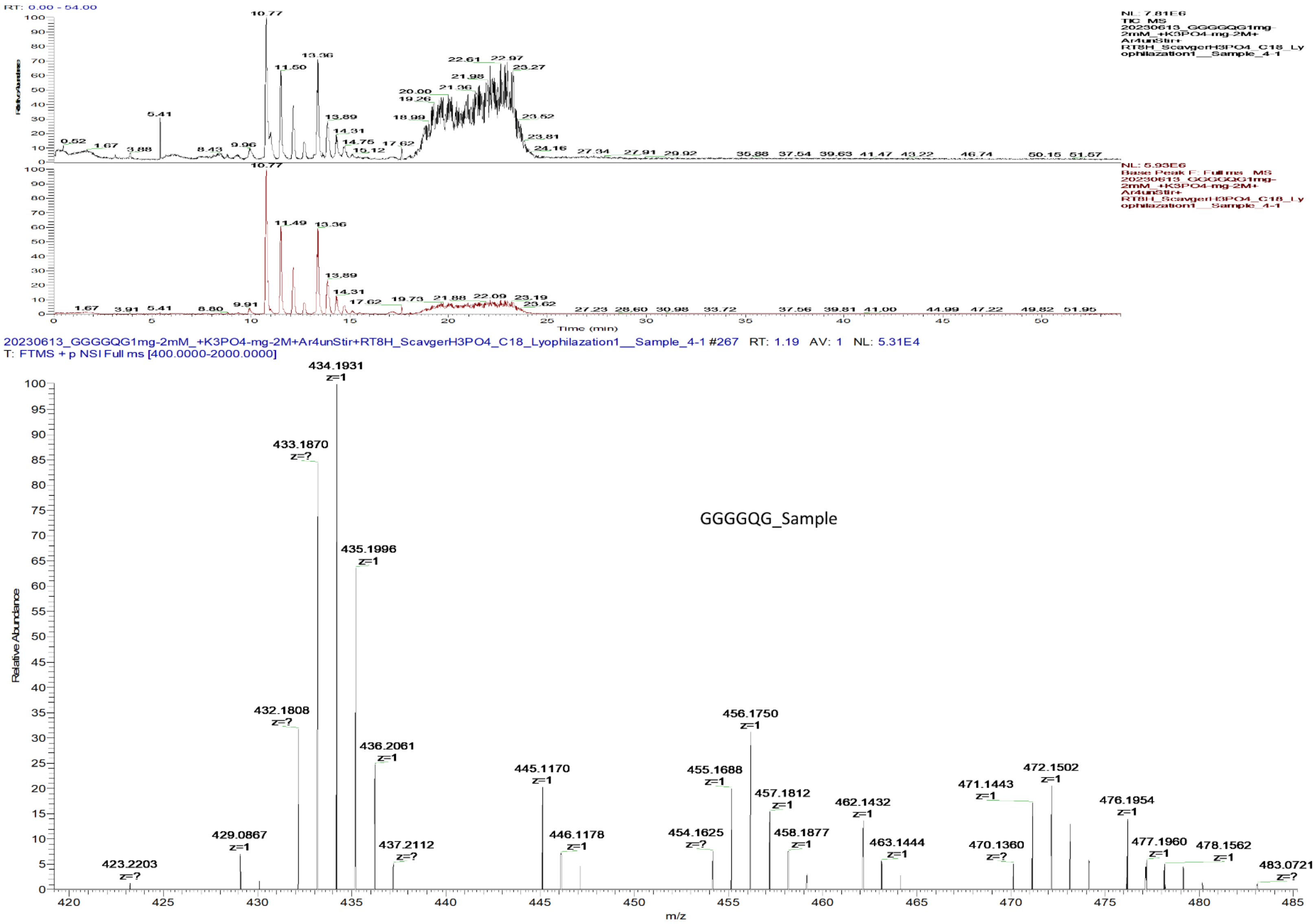
RT: 0.00 - 54.00
NL: 7.81E6
TIC MS
20230613_GGGGQG1mg-2mM_+K3PO4-mg-2M+Ar4unStir+RT8H_ScavgerH3PO4_C18_Lyophilazation1__Sample_4-1
NL: 5.93E6
Base Peak F: Full ms MS
20230613_GGGGQG1mg-2mM_+K3PO4-mg-2M+Ar4unStir+RT8H_ScavgerH3PO4_C18_Lyophilazation1__Sample_4-1
Time (min)
20230613_GGGGQG1mg-2mM_+K3PO4-mg-2M+Ar4unStir+RT8H_ScavgerH3PO4_C18_Lyophilazation1__Sample_4-1 #267 RT: 1.19 AV: 1 NL: 5.31E4
T: FTMS + p NSI Full ms [400.0000-2000.0000]
434.1931 z=1
433.1870 z=?
435.1996 z=1
432.1808 z=?
456.1750 z=1
436.2061 z=1
445.1170 z=1
455.1688 z=1
472.1502 z=1
471.1443 z=1
457.1812 z=1
462.1432 z=1
476.1954 z=1
429.0867 z=1
446.1178 z=1
454.1625 z=?
458.1877 z=1
463.1444 z=1
470.1360 z=?
477.1960 z=1
478.1562 z=1
437.2112 z=?
423.2203 z=?
483.0721 z=?
GGGGQG_Sample
Relative Abundance
m/z

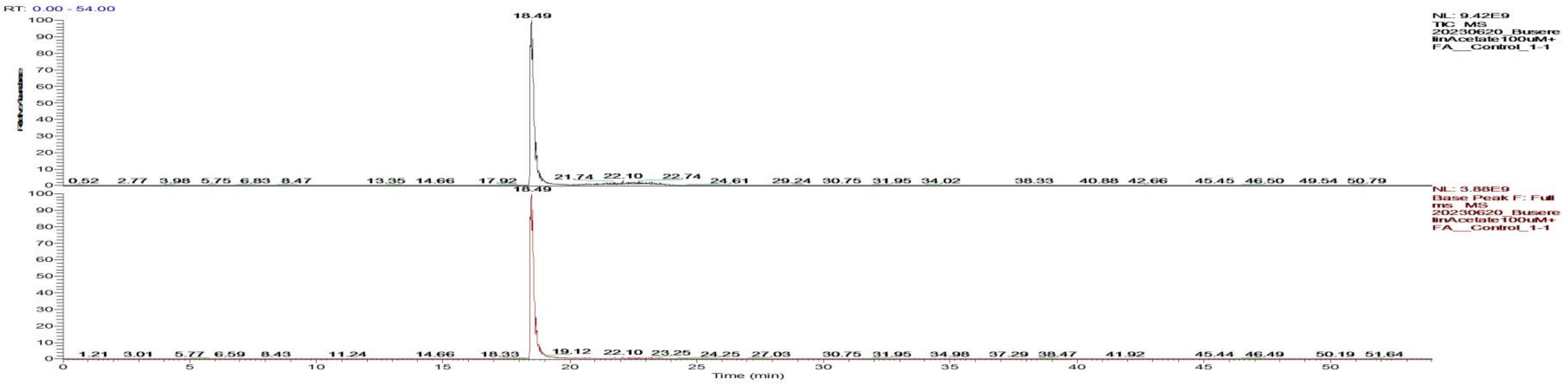
RT: 0.00 - 54.00
NL: 9.42E9
TIC MS
20230620_BuserelinAcetate100uM+FA__Control_1-1
NL: 3.88E9
Base Peak F: Full ms MS
20230620_BuserelinAcetate100uM+FA__Control_1-1
Relative Abundance
Time (min)

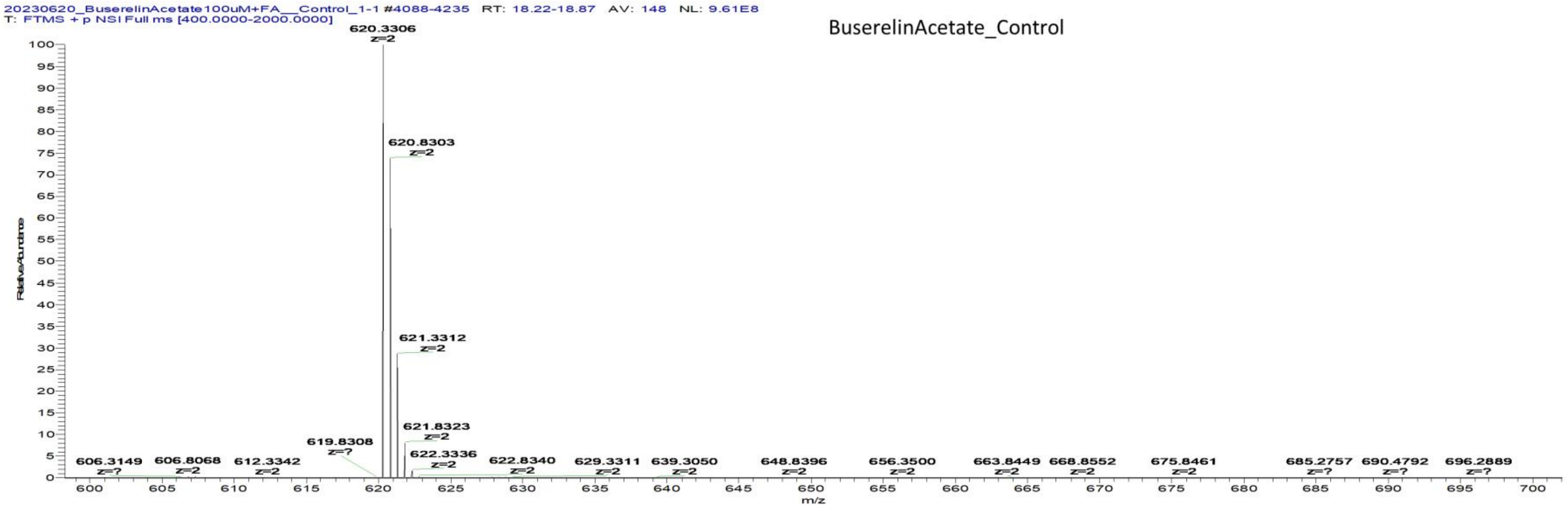
20230620_BuserelinAcetate100uM+FA__Control_1-1 #4088-4235 RT: 18.22-18.87 AV: 148 NL: 9.61E8
T: FTMS + p NSI Full ms [400.0000-2000.0000]
BuserelinAcetate_Control
620.3306
620.8303
621.3312
621.8323
622.3336
Relative Abundance
m/z

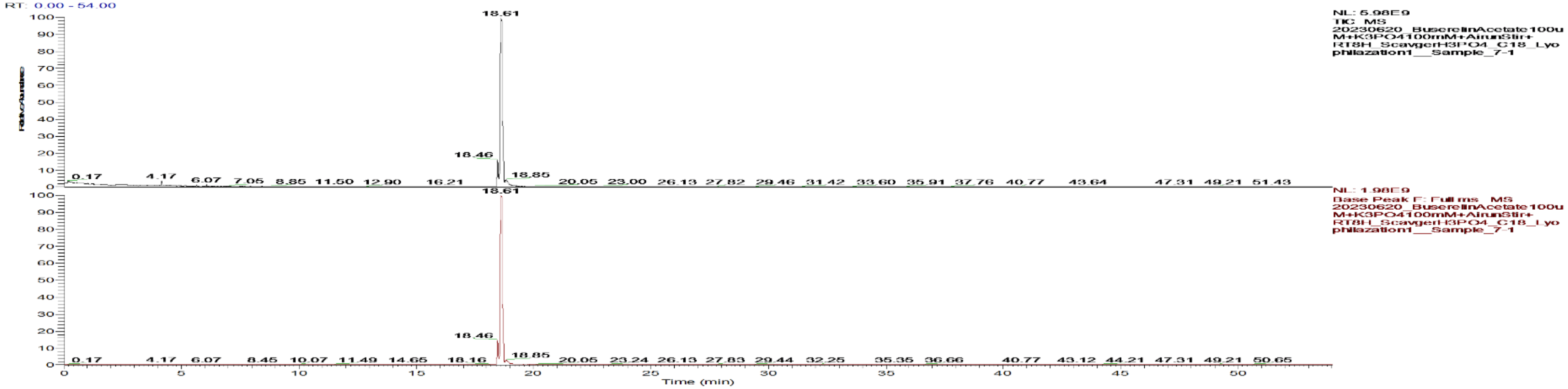
RT: 0.00 - 54.00
NL: 5.98E9
TIC MS
20230620_BuserelinAcetate100uM+K3PO4100mM+AirunStir+RT8H_ScavgerH3PO4_C18_Lyophilazation1__Sample_7-1
NL: 1.98E9
Base Peak F: Full ms MS
20230620_BuserelinAcetate100uM+K3PO4100mM+AirunStir+RT8H_ScavgerH3PO4_C18_Lyophilazation1__Sample_7-1
18.61
18.46
18.85
Time (min)
Relative Abundance

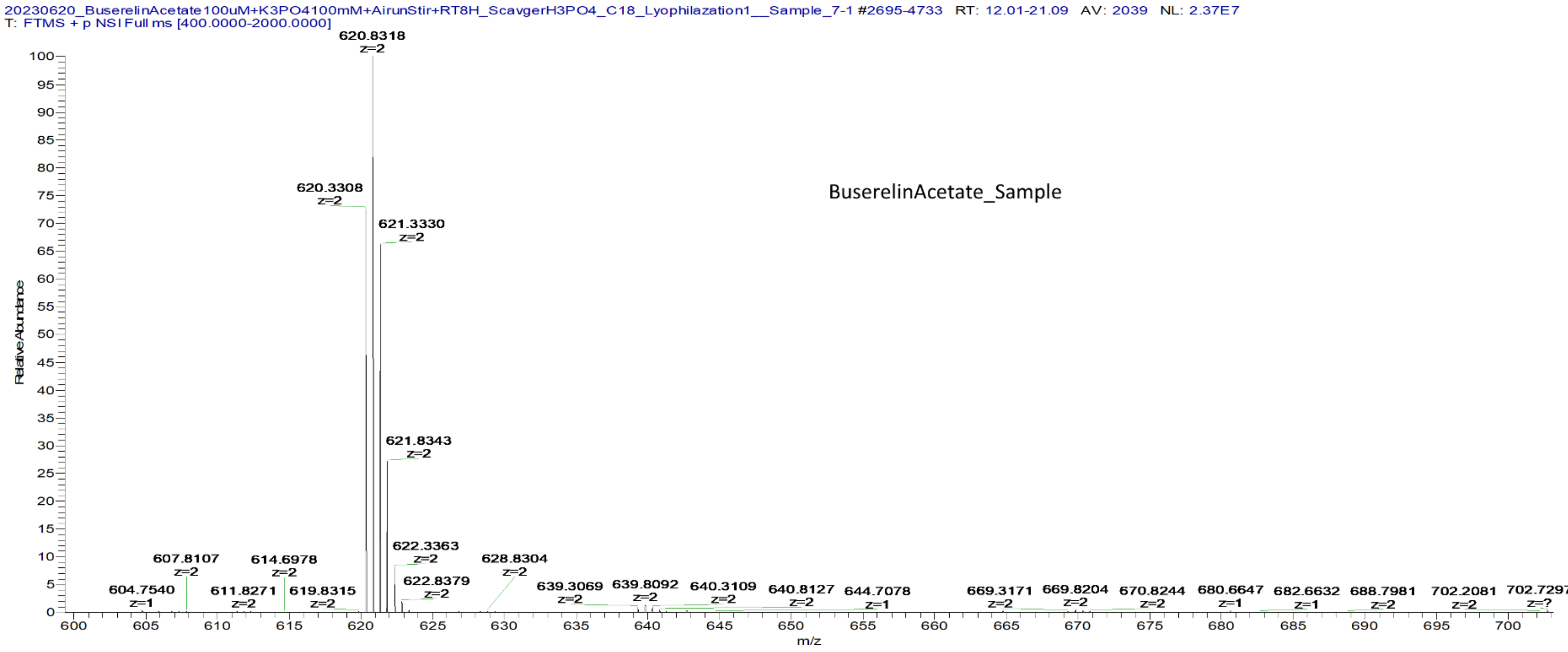
20230620_BuserelinAcetate100uM+K3PO4100mM+AirunStir+RT8H_ScavgerH3PO4_C18_Lyophilazation1__Sample_7-1 #2695-4733 RT: 12.01-21.09 AV: 2039 NL: 2.37E7
T: FTMS + p NSI Full ms [400.0000-2000.0000]
620.8318 z=2
620.3308 z=2
621.3330 z=2
621.8343 z=2
622.3363 z=2
622.8379 z=2
628.8304 z=2
607.8107 z=2
614.6978 z=2
604.7540 z=1
611.8271 z=2
619.8315 z=2
639.3069 z=2
639.8092 z=2
640.3109 z=2
640.8127 z=2
644.7078 z=1
669.3171 z=2
669.8204 z=2
670.8244 z=2
680.6647 z=1
682.6632 z=1
688.7981 z=2
702.2081 z=2
702.7297 z=?
BuserelinAcetate_Sample
Relative Abundance
m/z

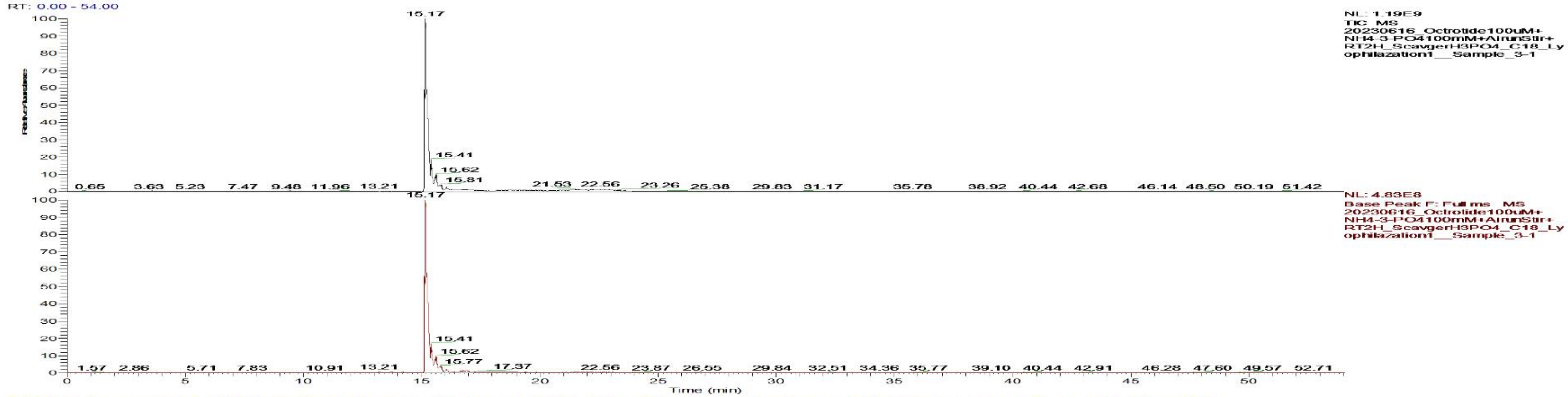
RT: 0.00 - 54.00
NL: 1.19E9
TIC MS
20230616_Octrotide100uM+NH4-3-PO4100mM+AirunStir+RT2H_ScavgerH3PO4_C18_Lyophilazation1__Sample_3-1
NL: 4.83E8
Base Peak F: Full ms MS
20230616_Octrotide100uM+NH4-3-PO4100mM+AirunStir+RT2H_ScavgerH3PO4_C18_Lyophilazation1__Sample_3-1
Time (min)
Relative Abundance
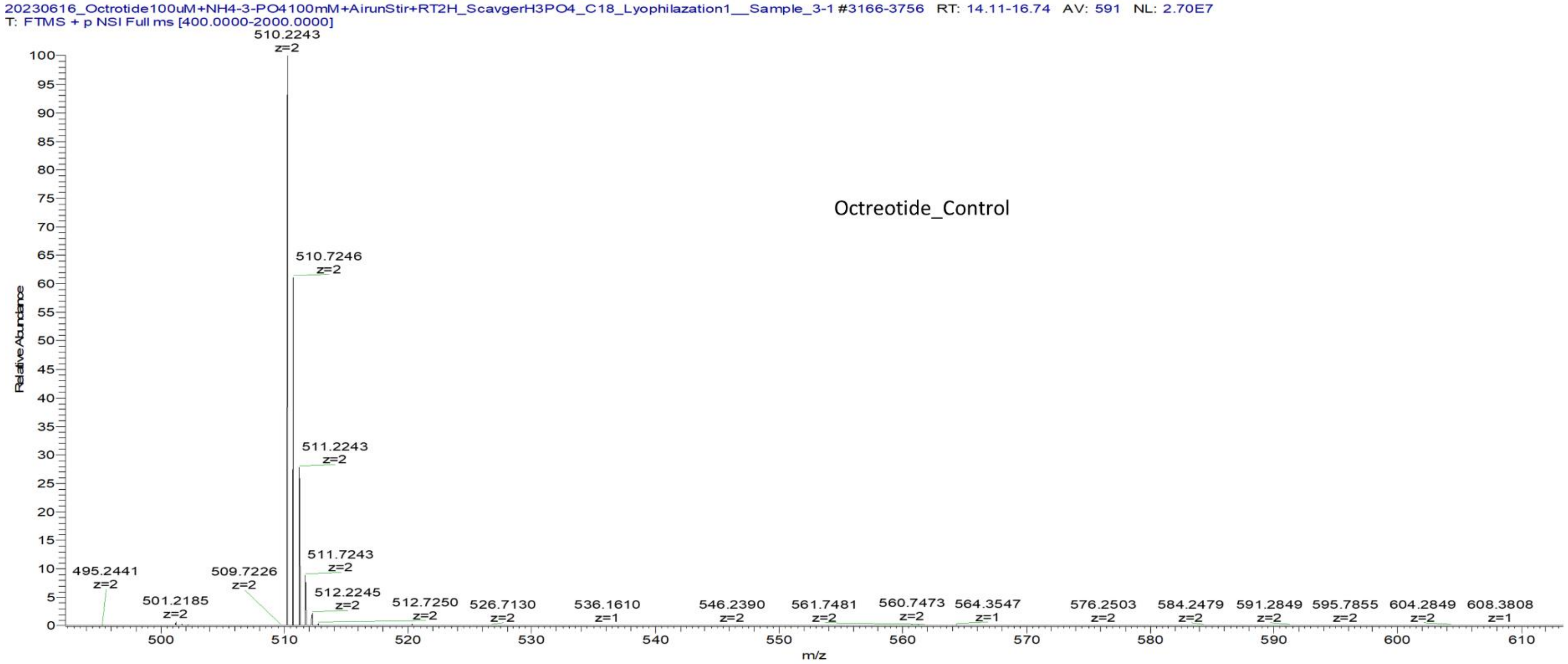
20230616_Octrotide100uM+NH4-3-PO4100mM+AirunStir+RT2H_ScavgerH3PO4_C18_Lyophilazation1__Sample_3-1 #3166-3756 RT: 14.11-16.74 AV: 591 NL: 2.70E7
T: FTMS + p NSI Full ms [400.0000-2000.0000]
Octreotide_Control
Relative Abundance
m/z

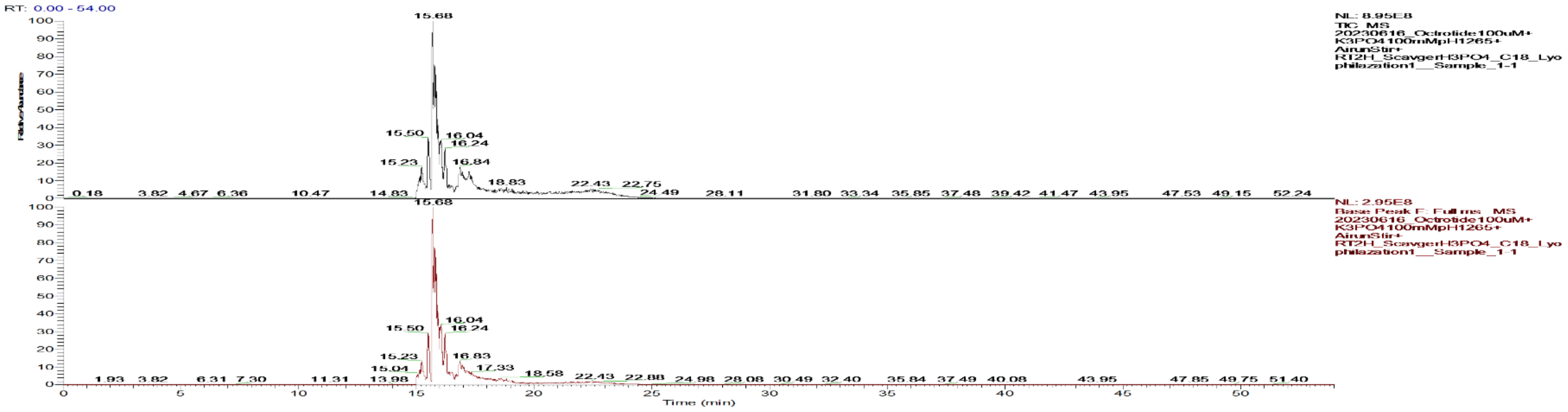

20230616_Octrotide100uM+K3PO4100mMpH1265+AirunStir+RT2H_ScavgerH3PO4_C18_Lyophilazation1__Sample_1-1 #2695-4733 RT: 12.01-21.09 AV: 2039 NL: 1.37E7
T: FTMS + p NSI Full ms [400.0000-2000.0000]

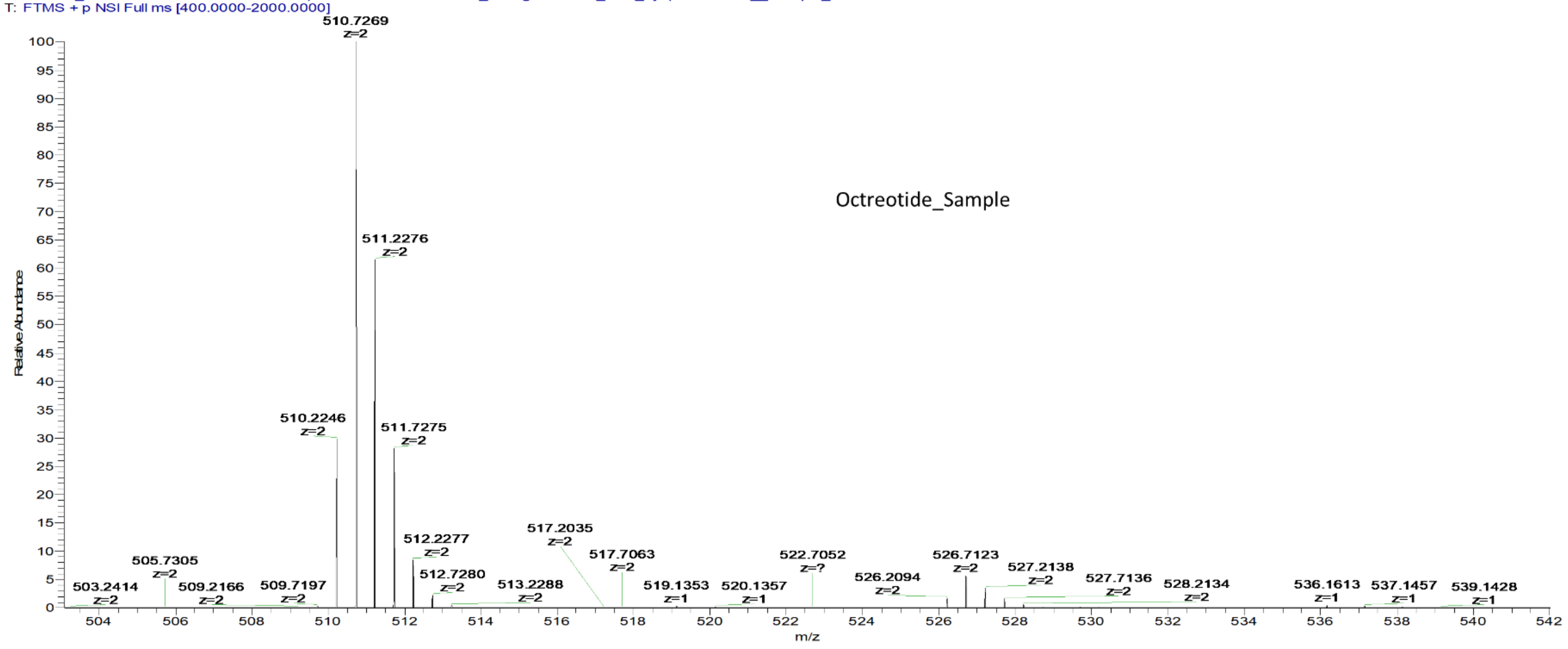

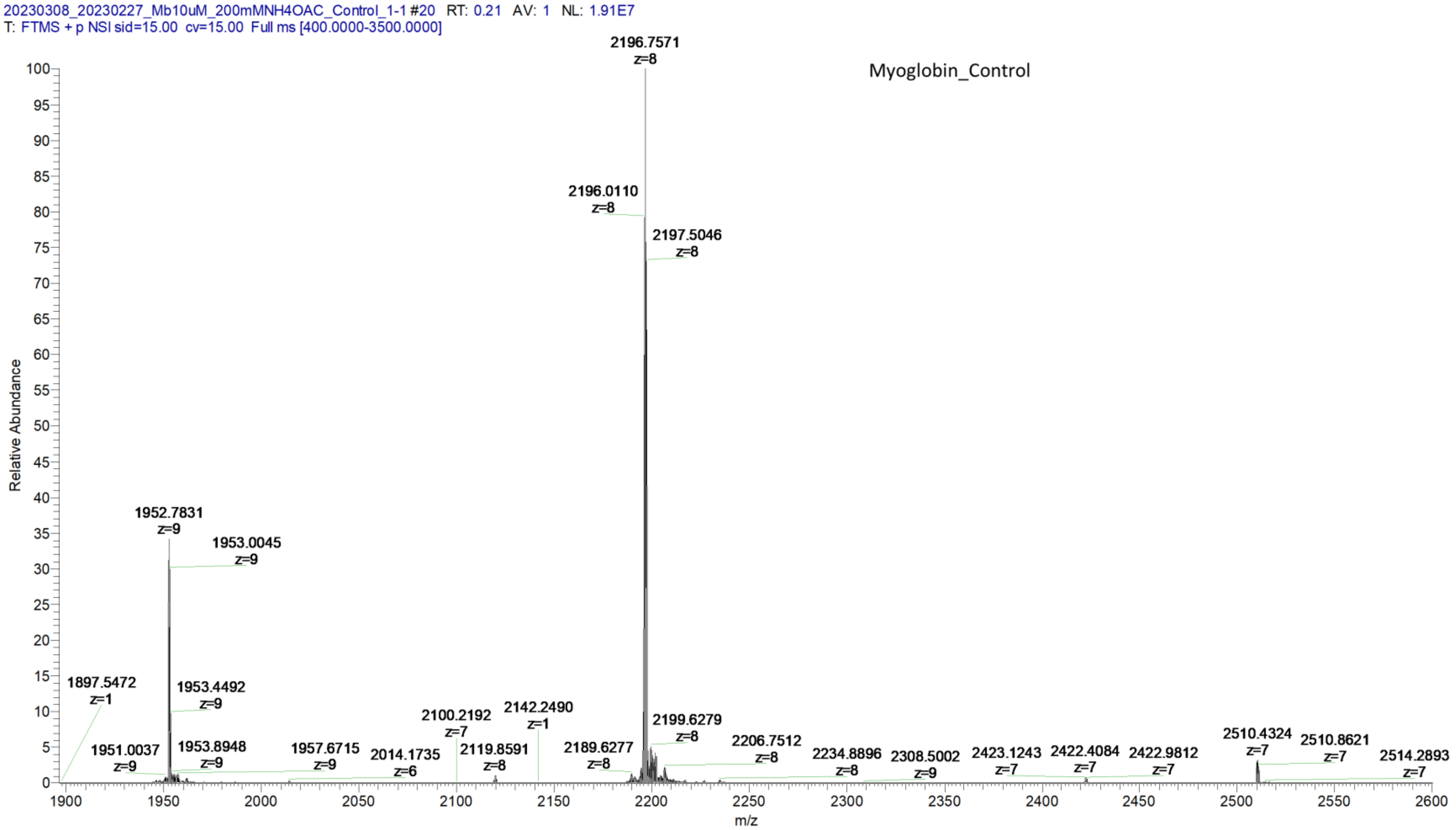

20230308_20230227_Mb10uM_200mMNH4OAC_Control_1-1 #20 RT: 0.21 AV: 1 NL: 1.91E7
T: FTMS + p NSI sid=15.00 cv=15.00 Full ms [400.0000-3500.0000]
Myoglobin_Control
Relative Abundance
m/z
2196.7571 z=8
2196.0110 z=8
2197.5046 z=8
1952.7831 z=9
1953.0045 z=9
1897.5472 z=1
1953.4492 z=9
1951.0037 z=9
1953.8948 z=9
1957.6715 z=9
2014.1735 z=6
2100.2192 z=7
2119.8591 z=8
2142.2490 z=1
2189.6277 z=8
2199.6279 z=8
2206.7512 z=8
2234.8896 z=8
2308.5002 z=9
2423.1243 z=7
2422.4084 z=7
2422.9812 z=7
2510.4324 z=7
2510.8621 z=7
2514.2893 z=7

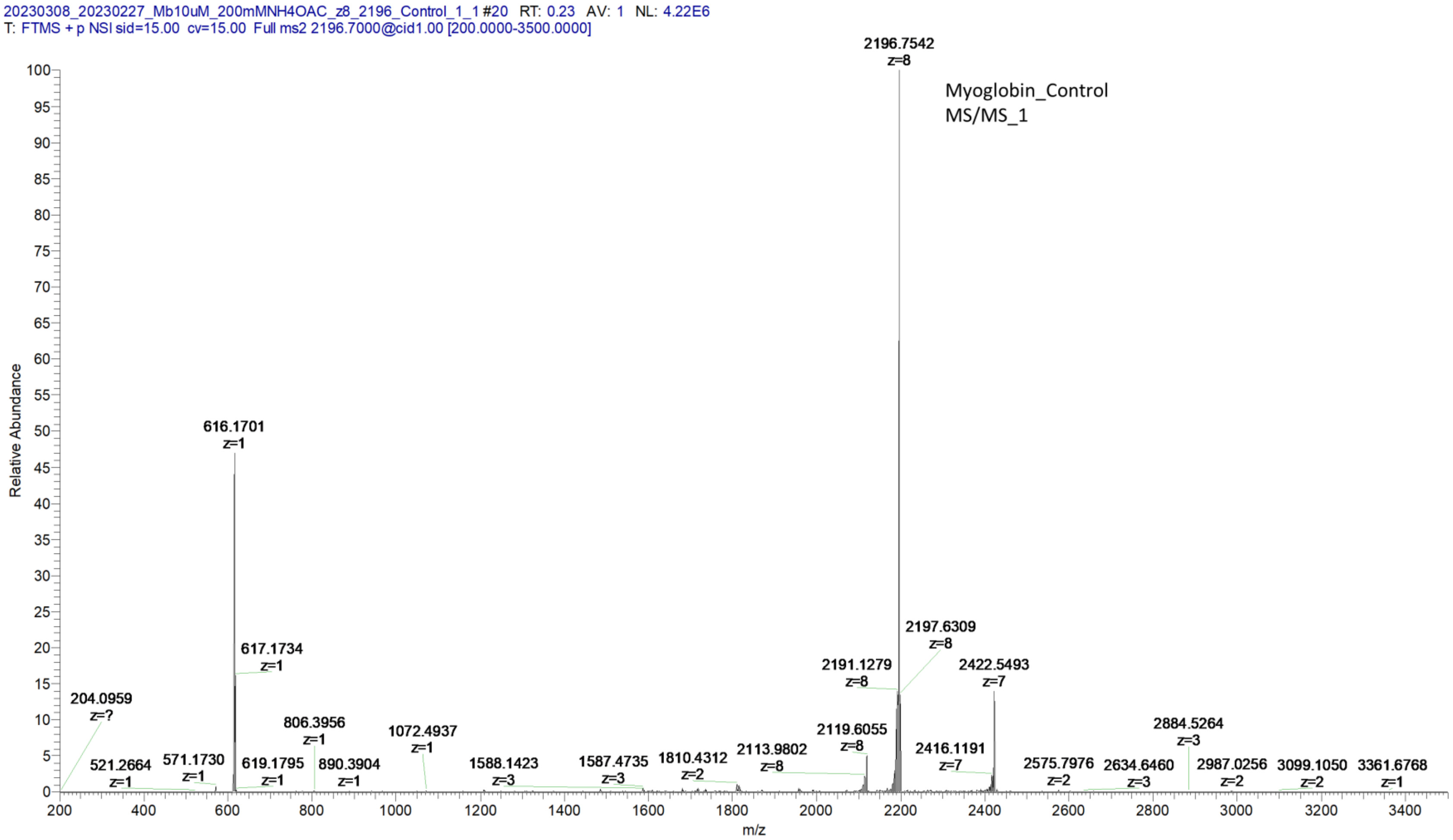
20230308_20230227_Mb10uM_200mMNH4OAC_z8_2196_Control_1_1 #20 RT: 0.23 AV: 1 NL: 4.22E6
T: FTMS + p NSI sid=15.00 cv=15.00 Full ms2 2196.7000@cid1.00 [200.0000-3500.0000]
Myoglobin_Control
MS/MS_1
Relative Abundance
m/z
2196.7542 z=8
616.1701 z=1
617.1734 z=1
204.0959 z=?
521.2664 z=1
571.1730 z=1
619.1795 z=1
806.3956 z=1
890.3904 z=1
1072.4937 z=1
1588.1423 z=3
1587.4735 z=3
1810.4312 z=2
2113.9802 z=8
2119.6055 z=8
2191.1279 z=8
2197.6309 z=8
2416.1191 z=7
2422.5493 z=7
2575.7976 z=2
2634.6460 z=3
2884.5264 z=3
2987.0256 z=2
3099.1050 z=2
3361.6768 z=1

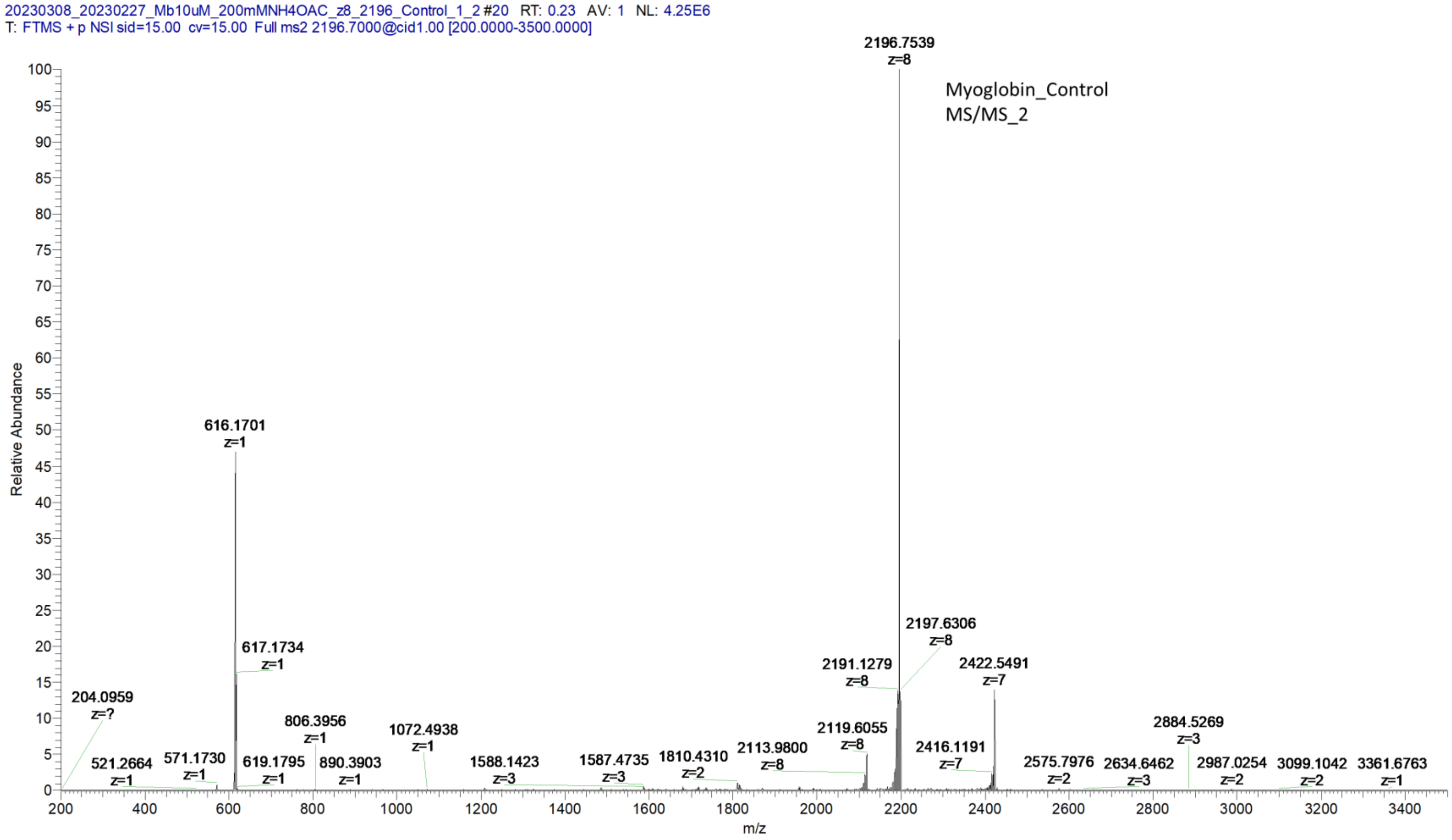
20230308_20230227_Mb10uM_200mMNH4OAC_z8_2196_Control_1_2 #20 RT: 0.23 AV: 1 NL: 4.25E6
T: FTMS + p NSI sid=15.00 cv=15.00 Full ms2 2196.7000@cid1.00 [200.0000-3500.0000]
Myoglobin_Control
MS/MS_2
2196.7539 z=8
616.1701 z=1
617.1734 z=1
2197.6306 z=8
2191.1279 z=8
2422.5491 z=7
204.0959 z=?
806.3956 z=1
1072.4938 z=1
2884.5269 z=3
2119.6055 z=8
2416.1191 z=7
521.2664 z=1
571.1730 z=1
619.1795 z=1
890.3903 z=1
1588.1423 z=3
1587.4735 z=3
1810.4310 z=2
2113.9800 z=8
2575.7976 z=2
2634.6462 z=3
2987.0254 z=2
3099.1042 z=2
3361.6763 z=1
Relative Abundance
m/z

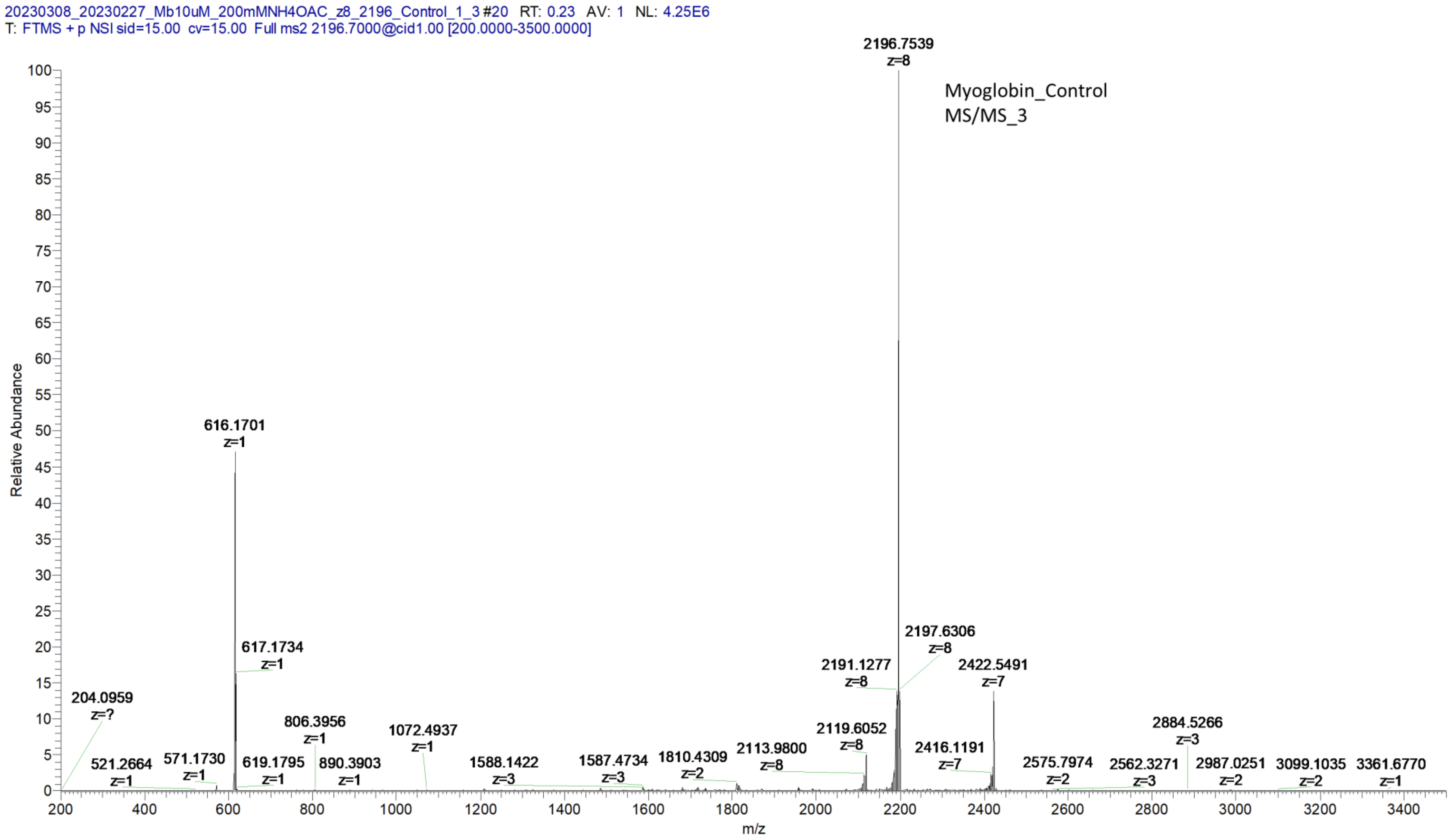
20230308_20230227_Mb10uM_200mMNH4OAC_z8_2196_Control_1_3 #20 RT: 0.23 AV: 1 NL: 4.25E6
T: FTMS + p NSI sid=15.00 cv=15.00 Full ms2 2196.7000@cid1.00 [200.0000-3500.0000]
Myoglobin_Control
MS/MS_3
Relative Abundance
m/z
2196.7539 z=8
616.1701 z=1
617.1734 z=1
2197.6306 z=8
2191.1277 z=8
2422.5491 z=7
204.0959 z=?
806.3956 z=1
1072.4937 z=1
2884.5266 z=3
2119.6052 z=8
2113.9800 z=8
2416.1191 z=7
1810.4309 z=2
521.2664 z=1
571.1730 z=1
619.1795 z=1
890.3903 z=1
1588.1422 z=3
1587.4734 z=3
2575.7974 z=2
2562.3271 z=3
2987.0251 z=2
3099.1035 z=2
3361.6770 z=1

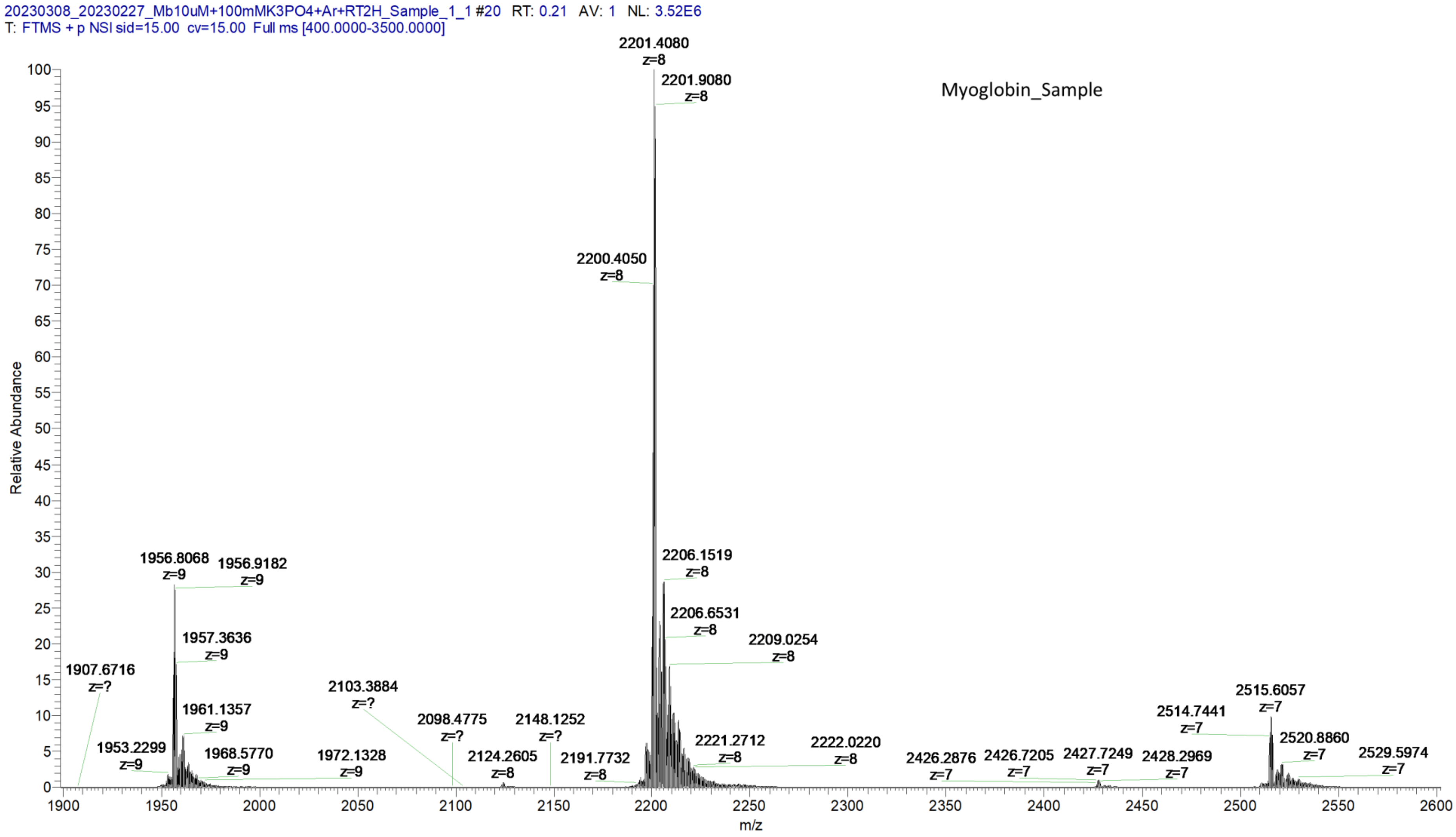
20230308_20230227_Mb10uM+100mMK3PO4+Ar+RT2H_Sample_1_1 #20 RT: 0.21 AV: 1 NL: 3.52E6
T: FTMS + p NSI sid=15.00 cv=15.00 Full ms [400.0000-3500.0000]
Myoglobin_Sample
2201.4080 z=8
2201.9080 z=8
2200.4050 z=8
2206.1519 z=8
2206.6531 z=8
2209.0254 z=8
2221.2712 z=8
2222.0220 z=8
1956.8068 z=9
1956.9182 z=9
1957.3636 z=9
1907.6716 z=?
1961.1357 z=9
1953.2299 z=9
1968.5770 z=9
1972.1328 z=9
2103.3884 z=?
2098.4775 z=?
2148.1252 z=?
2124.2605 z=8
2191.7732 z=8
2426.2876 z=7
2426.7205 z=7
2427.7249 z=7
2428.2969 z=7
2515.6057 z=7
2514.7441 z=7
2520.8860 z=7
2529.5974 z=7
Relative Abundance
m/z

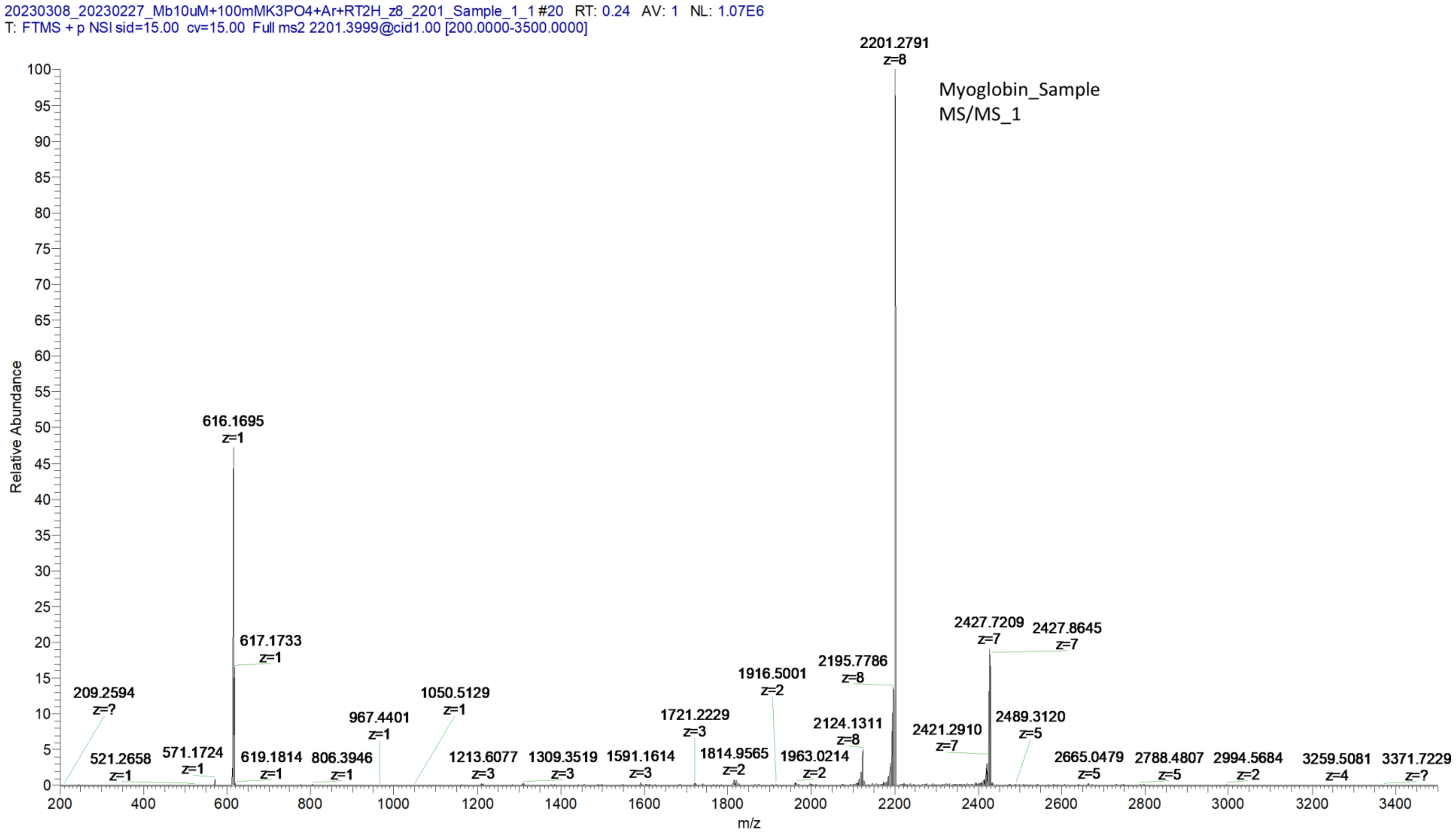

20230308_20230227_Mb10uM+100mMK3PO4+Ar+RT2H_z8_2201_Sample_1_1 #20 RT: 0.24 AV: 1 NL: 1.07E6
T: FTMS + p NSI sid=15.00 cv=15.00 Full ms2 2201.3999@cid1.00 [200.0000-3500.0000]
Myoglobin_Sample
MS/MS_1
Relative Abundance
m/z
2201.2791 z=8
616.1695 z=1
617.1733 z=1
209.2594 z=?
521.2658 z=1
571.1724 z=1
619.1814 z=1
806.3946 z=1
967.4401 z=1
1050.5129 z=1
1213.6077 z=3
1309.3519 z=3
1591.1614 z=3
1721.2229 z=3
1814.9565 z=2
1916.5001 z=2
1963.0214 z=2
2124.1311 z=8
2195.7786 z=8
2421.2910 z=7
2427.7209 z=7
2427.8645 z=7
2489.3120 z=5
2665.0479 z=5
2788.4807 z=5
2994.5684 z=2
3259.5081 z=4
3371.7229 z=?

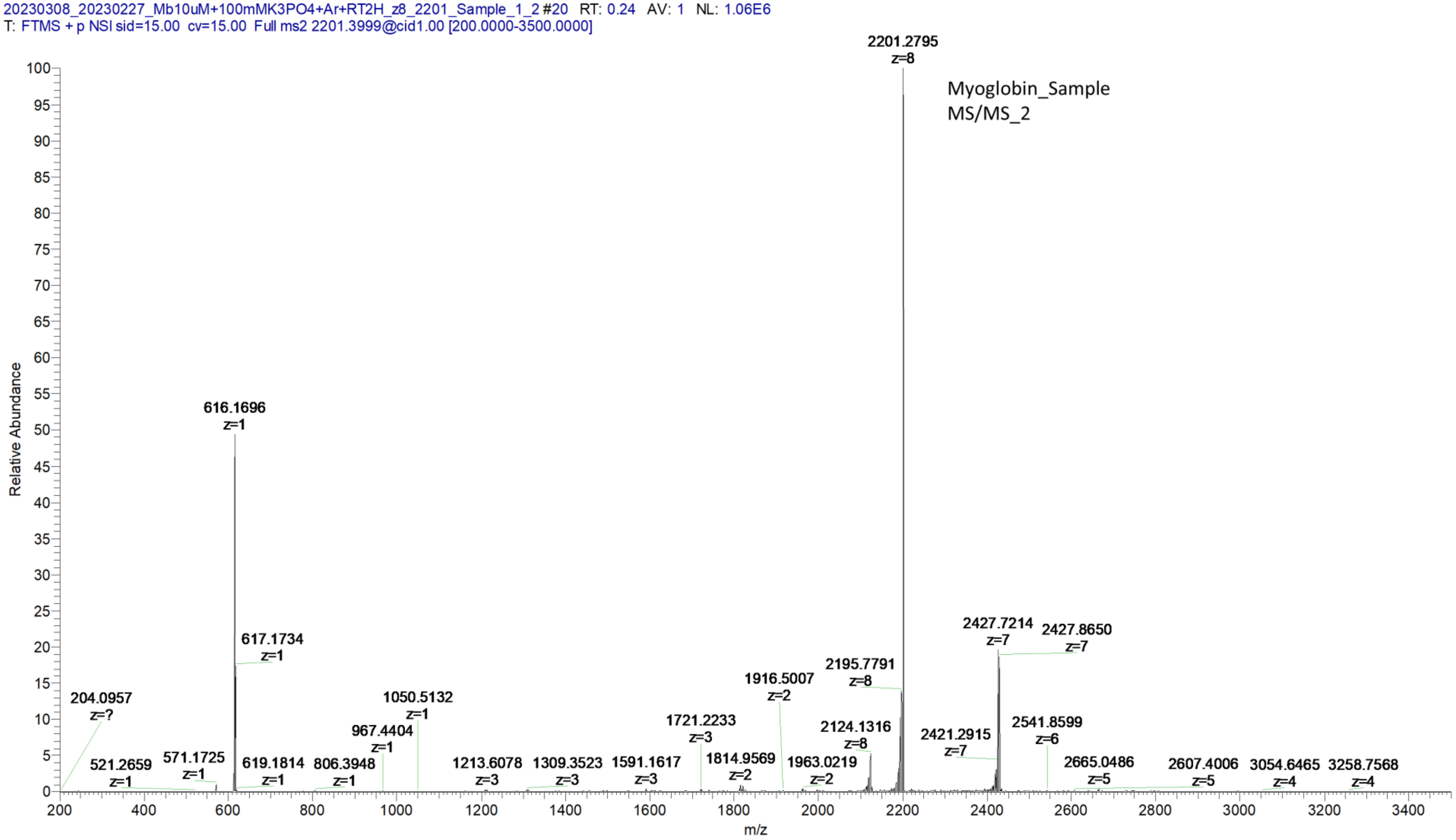
20230308_20230227_Mb10uM+100mMK3PO4+Ar+RT2H_z8_2201_Sample_1_2 #20 RT: 0.24 AV: 1 NL: 1.06E6
T: FTMS + p NSI sid=15.00 cv=15.00 Full ms2 2201.3999@cid1.00 [200.0000-3500.0000]
Myoglobin_Sample
MS/MS_2
2201.2795 z=8
616.1696 z=1
617.1734 z=1
204.0957 z=?
521.2659 z=1
571.1725 z=1
619.1814 z=1
806.3948 z=1
967.4404 z=1
1050.5132 z=1
1213.6078 z=3
1309.3523 z=3
1591.1617 z=3
1721.2233 z=3
1814.9569 z=2
1916.5007 z=2
1963.0219 z=2
2124.1316 z=8
2195.7791 z=8
2421.2915 z=7
2427.7214 z=7
2427.8650 z=7
2541.8599 z=6
2665.0486 z=5
2607.4006 z=5
3054.6465 z=4
3258.7568 z=4
Relative Abundance
m/z

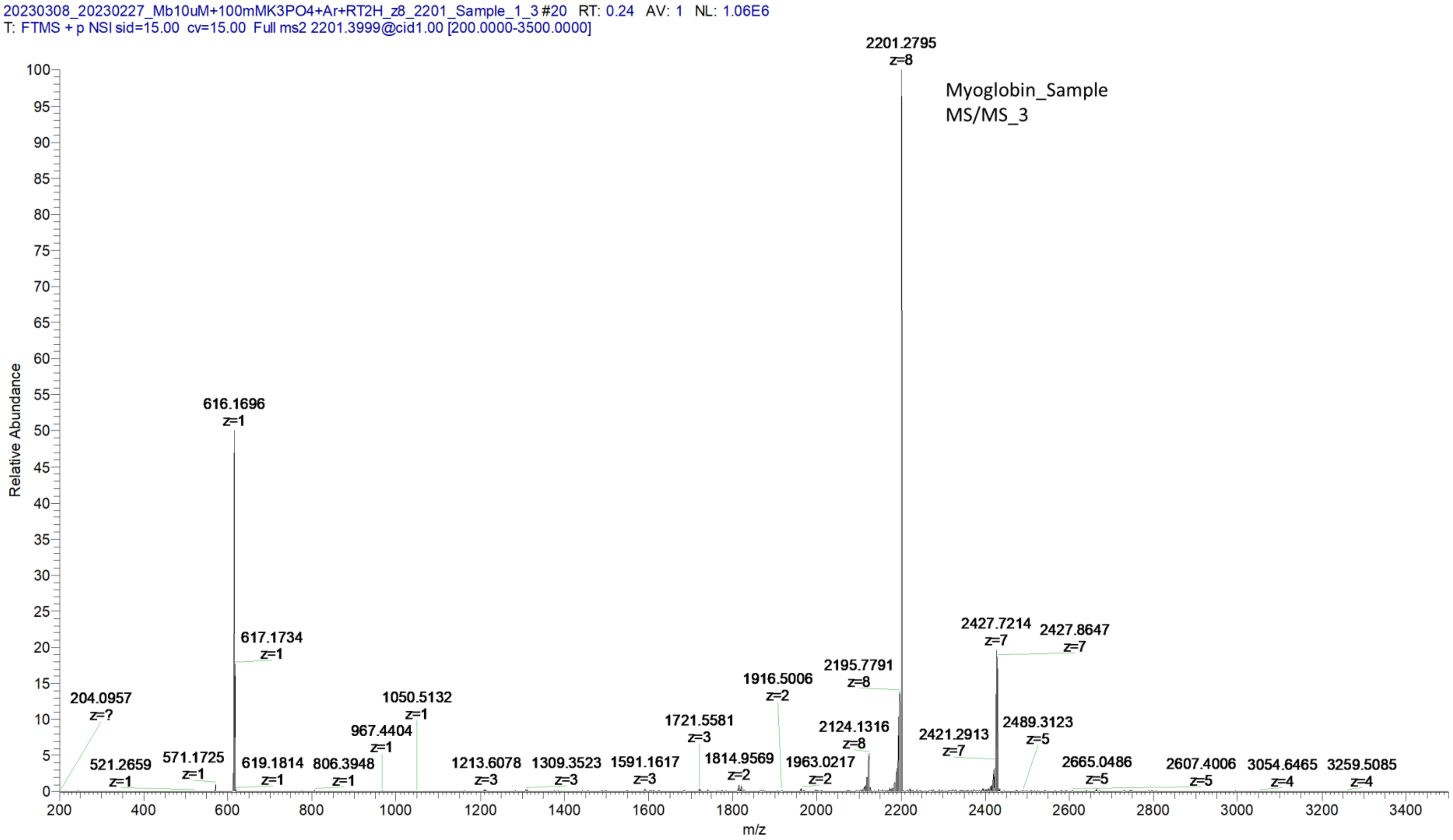
20230308_20230227_Mb10uM+100mMK3PO4+Ar+RT2H_z8_2201_Sample_1_3 #20 RT: 0.24 AV: 1 NL: 1.06E6
T: FTMS + p NSI sid=15.00 cv=15.00 Full ms2 2201.3999@cid1.00 [200.0000-3500.0000]
Myoglobin_Sample
MS/MS_3
Relative Abundance
m/z
2201.2795 z=8
616.1696 z=1
617.1734 z=1
204.0957 z=?
521.2659 z=1
571.1725 z=1
619.1814 z=1
806.3948 z=1
967.4404 z=1
1050.5132 z=1
1213.6078 z=3
1309.3523 z=3
1591.1617 z=3
1721.5581 z=3
1814.9569 z=2
1916.5006 z=2
1963.0217 z=2
2124.1316 z=8
2195.7791 z=8
2421.2913 z=7
2427.7214 z=7
2427.8647 z=7
2489.3123 z=5
2665.0486 z=5
2607.4006 z=5
3054.6465 z=4
3259.5085 z=4
